# Oyster Peptide-Ferrous Chelate Preparation Optimization Structural Characteristics and Enhanced Bioavailability

**DOI:** 10.3390/foods15020362

**Published:** 2026-01-20

**Authors:** Yijiu Zhang, Qi Yang, Ximing Yang, Shuzhen Cheng, Ming Du

**Affiliations:** 1 State Key Laboratory of Marine Food Processing & Safety Control, School of Food Science and Technology, Dalian Polytechnic University, Dalian 116034, China; 2 National Engineering Research Center of Seafood, School of Food Science and Technology, Dalian Polytechnic University, Dalian 116034, China; 3 Chaoyang Inspection and Testing Center, Chaoyang 122000, China; 4 College of Food Science and Engineering, Dalian Ocean University, Dalian 116023, China

**Keywords:** oyster peptide, ferrous chelate, preparation optimization, structural characterization, bioavailability, iron transport mechanism

## Abstract

Iron deficiency anemia remains a global nutritional challenge due to the low bioavailability and side effects of conventional inorganic iron supplements. A novel organic iron supplement, oyster peptide ferrous chelate (OP-Fe), was prepared using oyster peptides as ligands. Its preparation process was optimized via single-factor experiments and statistical methods with the optimal conditions identified as 1% peptide concentration, 35 °C chelation temperature, 3.91:1 peptide-to-iron ratio, 1.49% ascorbic acid concentration and pH 6.05. Under these conditions, the chelate’s iron content reached 15.44 ± 0.18 g/kg. Multi-dimensional characterization confirmed that Fe^2+^ formed stable complexes with oyster peptides through carboxyl, amino, and imidazole groups. In vitro Caco-2 cell experiments showed OP-Fe achieved a maximum iron absorption rate of 76.07%, significantly higher than ferrous sulfate (52.39%). In vivo pharmacokinetic studies in mice demonstrated higher iron accumulation in serum and small intestine for OP-Fe. Key iron transport-related genes (*PEPT1*, *TFR1*, *DMT1*) were upregulated, contributing to enhanced absorption. OP-Fe exhibits favorable structural stability and bioavailability, holding potential as an efficient iron supplement.

## 1. Introduction

Iron is an essential trace element in the human body. As a vital component of myoglobin, hemoglobin, cytochromes, and various enzymes, it is critical for oxygen transport, electron transfer, and oxidoreductase activity. However, its typically poor absorption and utilization contribute to iron deficiency disorders, with anemia constituting a significant global public health and nutritional issue [1,2]. Around one-third of individuals worldwide are estimated to suffer from anemia, the most widespread type of which is caused by insufficient iron levels [3].

Common oral iron supplements are predominantly formulated as ferrous salts, including ferrous sulfate, ferrous gluconate, and ferrous lactate [4]. Their clinical application, however, is often limited by drawbacks such as low bioavailability, gastric mucosal irritation, chemical instability, and potential antagonistic interactions with other metal ions [5,6]. Consequently, there is a pressing demand to design advanced iron supplementation options that offer improved effectiveness with minimized adverse reactions [7]. The conversion of iron from its inorganic form to an organic form has been proven as an effective strategy to enhance its bioavailability [8]. Among various novel iron supplements, peptides are considered ideal iron binding ligands due to their coordination capable side chain groups and suitable spatial conformation, which enable them to form stable complexes with iron. In recent years, iron-binding peptides from food protein hydrolysates have become important for developing new iron supplements. This is because they offer benefits like high bioavailability, good solubility, and low stomach irritation. For example, peptides from Antarctic krill [9,10] contain a lot of aspartic acid and glutamic acid. Their side-chain carboxyl groups can bind iron well through chelation and stay soluble at neutral pH. Iron-binding peptides from chickpea protein [11] show clear antioxidant activity. This helps lower oxidative damage during iron absorption. Peptides from whey protein [12] have very good intestinal mucosal permeability and absorption efficiency, so they help iron become absorbed and transported. Mung bean peptides [13] can also form stable complexes with iron using certain sequences that contain residues like histidine and cysteine, which improves iron bioaccessibility [14].

Research indicates that marine proteins represent a high quality source of human nutrition. Oyster, a highly valuable marine shellfish, possesses considerable edible and medicinal value [15]. It is an important source of high-quality animal protein, rich in essential amino acids, and offers nutritional advantages over terrestrial proteins [16]. Notably, oyster peptides contain abundant iron binding sites. Current research on oyster peptides has largely focused on their chelation capacity with zinc ions. Existing studies have shown that the secondary structural features of oyster peptide-iron complexes are associated with their higher in vivo absorption efficiency, which surpasses that of ferrous sulfate [17]. While most related studies concentrate on bioavailability assessment, the specific mechanisms underlying their absorption pathways remain unclear. In this study, an oyster peptide ferrous chelate (OP-Fe) was successfully synthesized through an optimized preparation process. Its structural characteristics and bioavailability were systematically analyzed to determine the optimal chelation conditions and binding sites. Furthermore, the differences in the absorption pathways of this chelate were investigated, providing a theoretical foundation for its development and application as a novel iron supplement.

## 2. Materials and Methods

### 2.1. Materials

The oyster peptide sample was purchased from Dezhou Lan li Biotechnology Co., Ltd. (Dezhou, China). The FastKing gDNA removal and first-strand cDNA synthesis premix kit, along with the SuperReal PreMix Plus (SYBR Green) kit, were purchased from Tiangen Biochemical Technology Co., Ltd. (Beijing, China). The Eastep^TM^ Super Total RNA Extraction Kit was obtained from Promega Biotechnology Co., Ltd. (Beijing, China). Caco-2 cell culture medium was supplied by Procell Life Science & Technology Co., Ltd. (Wuhan, China). FeCl_2_·4H_2_O and FeSO_4_ were obtained from Shanghai Macklin Biochemical Technology Co., Ltd. (Shanghai, China). All other reagents and chemicals used were of analytical grade.

### 2.2. Preparation and Iron Content Determination of the Oyster Peptide-Ferrous Chelate

A single-factor experimental design was used to study the effects of oyster peptide concentration (1%, 2%, 3%, 4%, 5%), ascorbic acid concentration (0.2%, 0.6%, 1%, 1.4%, 1.8%), pH (4, 5, 6, 7, 8), chelation temperature (25 °C, 35 °C, 45 °C, 55 °C, 65 °C), and peptide-to-iron ratio (1:1, 2:1, 3:1, 4:1, 5:1) on the iron chelating capacity. Oyster peptides were dissolved in water, and ascorbic acid was added. The pH of the solution was then adjusted. Subsequently, FeCl_2_·4H_2_O was added proportionally and mixed thoroughly. Following incubation under controlled thermal conditions for a predetermined duration, the mixture was subjected to dialysis using a membrane with a 200 Da molecular weight cutoff over a 24 h period. After completion of dialysis, the resulting material was collected [18].

The obtained chelates were pre-frozen at −50 °C for 2 h in a vacuum freeze-dryer, followed by freeze-drying for 24 h with a shelf temperature of 50 °C. To release the iron ions from the chelates into their free inorganic ferrous form, the freeze-dried samples were subjected to high-temperature digestion with nitric acid. The sample is digested in a digestion furnace at 220 °C. The digested samples were then diluted to a final volume of 25 mL. Finally, the iron content of the chelates was determined using an atomic absorption spectrophotometer (AAS, Hitachi, Japan). The iron content was calculated according to Formula (1).

(1) $$Iron\;content\;of\;the\;chelate\;(g/kg)=\frac{C\times V_{0}}{M\times 1000}$$

where

*C*—Concentration converted from the standard curve (mg/L);

*V*_0_—Final volume after dilution (mL);

*M*—Mass of the weighed sample (g).

### 2.3. Response Surface Optimization and Preparation of Chelate

Based on the single-factor experimental results, three key factors significantly influencing the iron content of the chelate peptide to ferrous chloride mass ratio (1:1, 3:1, 5:1), pH (4, 6, 8), and ascorbic acid concentration (0.2%, 1%, 1.8%) were selected for the optimization of oyster peptide-iron chelate preparation using response surface methodology. A three-factor, three-level experimental design comprising 15 runs with 3 replicates at the center point was employed. A second-order polynomial regression model was constructed to characterize the association between the dependent variable and the independent factors [19].

(2) $$Y=K_{0}+\Sigma K_{i}x_{i}+\Sigma K_{ii}{xi}^{2}+\Sigma \Sigma K_{ij}x_{i}x_{j}$$

In the model, *Y* represents the predicted response value, *K*_0_ is the intercept term, *K_i_* denotes the linear coefficients, *K_ii_* signifies the quadratic coefficients, and *K_ij_* stands for the interaction coefficients, where *x_i_* and *x_j_* are the coded independent variables.

### 2.4. Characterization of the Oyster Peptide-Ferrous Chelate

#### 2.4.1. Fourier-Transform Infrared Spectroscopy (FTIR)

OP and OP-Fe samples were characterized using an FTIR spectrophotometer (Nicolet-20DXB, Thermo Fisher Scientific, Waltham, MA, USA) with the KBr pellet method. Lyophilized samples (1 mg) were mixed with 100 mg of dried KBr, ground thoroughly, and pressed into transparent pellets. Spectra were recorded over a wavenumber range of 4000 to 500 cm^−1^ with a resolution of 4 cm^−1^.

#### 2.4.2. Circular Dichroism Spectroscopy (CD)

The method was adapted from Li’s [20] experimental protocol. Circular dichroism (CD) spectra of OP and OP-Fe were recorded using a CD spectrophotometer (J-1500, JASCO, Tokyo, Japan) over a wavelength range of 190–260 nm. OP and OP-Fe powder samples were, respectively, dissolved in deionized water to prepare 5 mL solutions with a concentration of 0.5 mg/mL each and analyzed at 1 nm intervals [21]. Measurements were performed at room temperature (approximately 25 °C) using a 1 mm quartz cuvette purged with nitrogen.

#### 2.4.3. Fluorescence Spectroscopy (FL)

OP and OP-Fe powder samples were, respectively, dissolved in deionized water to prepare two solutions, each with a concentration of 0.05 mg/mL and a volume of 10 mL. Fluorescence spectra of OP and OP-Fe were obtained using a Hitachi F-2700 spectrofluorometer (Hitachi Ltd., Tokyo, Japan). The excitation wavelength was set at 285 nm, and the emission spectra were scanned from 280 to 450 nm. The slit widths were 10 nm, and the photomultiplier tube voltage was maintained at 700 V [22].

#### 2.4.4. X-Ray Diffraction (XRD)

The crystalline structures of OP and OP-Fe were analyzed using an X-ray diffractometer (XRD-7000S, Shimadzu, Kyoto, Japan). Approximately 0.5 g of sample, sieved through a 300-mesh screen to obtain fine powder, was placed into a sample holder. Continuous scanning was performed over a 2θ range of 5° to 70° at a scanning speed of 5°/min.

#### 2.4.5. Molecular Docking

The peptide models serving as the docking receptor were derived from the molecular library of oyster peptides (Table S1). Molecular docking was performed using the CDOCKER protocol within the Discovery Studio 2019 Client software (DS 2019 Client, Biovia, San Diego, CA, USA), with Fe^2+^ defined as the small-molecule ligand and the peptides from the library as receptors. Peptides were screened based on their—CDOCKER ENERGY scores, where a value greater than 30 indicates a relatively high binding energy between the receptor and ligand. Four peptides with—CDOCKER ENERGY scores exceeding 30 were selected for further analysis [23].

### 2.5. Investigation of Iron Absorption Rate in Caco-2 Cell Monolayers

Prior to the formal experiments, a cytotoxicity test was performed using the CCK-8 reagent. In this study, Caco-2 cells were used as an intestinal epithelial barrier model, as described by Jantarajit [24]. Specifically, Caco-2 cells were seeded at a density of 1 × 10^5^ cells/mL onto 12-well Transwell plates (polyester membrane, pore size 0.40 μm, diameter 12 mm) and cultured for 21 days. Beginning 24 h after seeding, the culture medium was replaced daily, with 0.5 mL added to the apical (AP) compartment and 1.5 mL to the basolateral (BL) compartment. During the 21-day culture period, transepithelial electrical resistance (TEER) and the apical to basolateral alkaline phosphatase activity ratio (AKP, AP/BL) were monitored periodically.

Prior to experimental treatment, the cell inserts were washed twice with HBSS (pH 7.4). Then, 0.5 mL of either OP, OP-Fe, or FeSO_4_ solution was applied to the AP side and incubated for 10 min, 30 min, 2 h, 6 h, or 24 h. During incubation, the Transwell plates were maintained at 37 °C in a 5% CO_2_ atmosphere. Following incubation, cells and the solution from the lower chamber were collected, and the ferrous iron concentration was measured using flame atomic absorption spectroscopy. All experiments were conducted in triplicate. The iron absorption rate was calculated according to Equation (3).

(3) $$Absorption\;rate\left(\%\right)=\frac{M_{cell}+M_{BL}}{M_{AP}}$$

where

*M_cell_*—the mass of ferrous iron within the cells (μg);

*M_BL_*—the mass of ferrous iron on the basolateral (*BL*) side (μg);

*M_AP_*—the mass of the sample added to the apical (*AP*) side (μg).

### 2.6. Iron Deficiency Animal Models and Histological Analysis

The animal experiments were approved by the Institutional Animal Care and Use Committee of Dalian Polytechnic University (Approval Date 28 April 2024, DLPU2024016). For the animal experiments, Six-week-old male C57 mice were obtained from Liaoning Changsheng Biotechnology Co., Ltd (Dalian, China). All mice were housed under standard laboratory conditions, with the ambient temperature maintained at 23 ± 2 °C, relative humidity at 55 ± 5%, and a 12 h light/dark cycle. Throughout the study, the mice had free access to food and water.

A total of 33 mice were prepared and divided into three groups [25]: control group (A), OP-Fe group (B), and FeSO_4_ group (C).

The control group (*n* = 3) received neither OP-Fe nor FeSO_4_. In the OP-Fe group, three mice formed one subgroup and were administered OP-Fe by gavage at time points of 5 min, 10 min, 30 min, 1 h and 2 h. The FeSO_4_ group was set up in the same manner, with three mice per subgroup receiving FeSO_4_ gavage following the same time schedule as group B. All mice were tail-marked for identification. If the mice exhibited signs of distress during the gavage procedure, they would be euthanized. The dose of both compounds was 10 mg/kg. After administration, blood and small intestine samples were collected. Blood samples were centrifuged at 1500 rpm for 15 min to obtain serum. The intestinal tissues were digested with nitric acid. Both the digested intestinal samples and the serum were analyzed for iron content using atomic absorption spectrophotometry. The results were compared with data from the control group to evaluate the effects of OP-Fe and FeSO_4_ on iron levels in the small intestine and serum.

### 2.7. qRT-PCR Analysis

The method was adapted from Yang’s [25] experimental protocol with modifications. Cells were seeded at a density of 1 × 10^6^ cells per well and cultured until fully differentiated. Subsequently, the cells were treated with either OP-Fe or FeSO_4_ for 10 min, 30 min, 2 h, 6 h, and 24 h. Following treatment, total RNA was extracted and reverse transcribed into cDNA. Gene expression analysis was conducted through real-time quantitative polymerase chain reaction, with GAPDH serving as the endogenous control gene. Normalized expression values were derived using the comparative quantification (2^−ΔΔCT^) method according to established protocols.

### 2.8. Statistical Analysis

Statistical analysis was performed using SPSS (Version 16.0). For significant main effects, means were considered significantly different at the 0.05 probability level. Data are expressed as the mean of three replicates. Graphs were generated using GraphPad Prism (version 10.4.2). Molecular docking was performed using Discovery Studio 2019 Client.

## 3. Results and Discussion

### 3.1. Preparation Process of the OP-Fe Chelate

**Figure 1:**
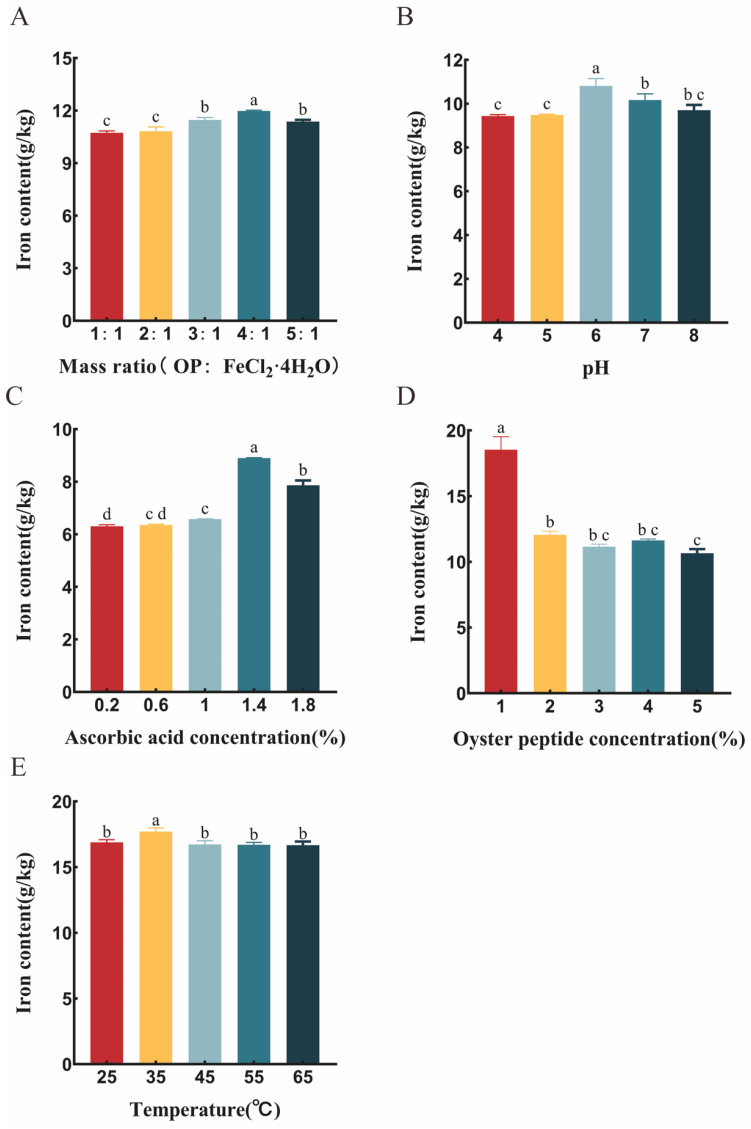
The single-factor experiments of the chelation between oyster peptide (OP) and ferrous ions. The effects of the following factors on the iron content of the oyster peptide ferrous chelate are presented. (**A**) peptide-to-iron mass ratio (OP to FeCl_2_·4H_2_O, *w*/*w*), (**B**) pH, (**C**) ascorbic acid concentration, (**D**) peptide concentration and (**E**) temperature. The data were analyzed by one-way ANOVA. All data are expressed as average values and standard deviations based on three independent replicates (*n* = 3). Statistically significant differences (*p* < 0.05) among groups are denoted by distinct lowercase superscript letters.

Based on the effect of peptide-to-iron mass ratio on chelation efficiency shown in Figure 1A, the experiment started at a 1:1 ratio. It was found that as the amount of oyster peptide (OP) increased, the iron content of the chelate showed a steady upward trend. When the peptide-to-iron mass ratio reached 4:1, the iron content peaked at 11.98 ± 0.45 g/kg. However, beyond this ratio, further increasing in OP led to a decrease in the iron content of the chelate. This indicates that the efficiency of Fe^2+^ binding per unit mass of OP decreased. The possible reason is that a high concentration of peptide molecules increases steric hindrance in the solution, reducing the chance of contact between metal ions and binding sites. In high-concentration systems, peptide chains undergo self-association driven by non-covalent intermolecular interactions such as hydrophobic effects, hydrogen bonding and electrostatic attraction. This leads to the assembly of soluble aggregates or microgels [26]. Such aggregation behavior results in a dual inhibitory effect, on the one hand, through steric shielding, metal binding groups originally exposed become buried within the core of the aggregates, reducing the solvent accessibility of the binding sites. On the other hand, it induces a conformational shift in the peptide chains from an extended to a compact folded state [27]. This rearrangement alters the spatial orientation of key ligand atoms, such as the imidazole nitrogen of histidine and the carboxyl oxygen of glutamate, thereby disrupting the optimal geometry required for forming stable coordination bonds with metal ions []. Simultaneously, an excess of OP molecules intensifies competition for Fe^2+^, dispersing the binding capacity of individual peptide molecules and thus lowering the final iron content [21].

For the coordination reaction conditions, Figure 1B illustrates the effect of pH at a fixed peptide-to-iron mass ratio of 2:1. The results showed that pH 7 was the optimal reaction condition, yielding the highest iron content in the chelate (10.81 ± 0.32 g/kg). In acidic environments (pH < 6), a large number of H^+^ ions competitively occupy the negatively charged amino acid binding sites on OP, hindering iron binding. Therefore, the iron content increased with rising pH. When the pH exceeded 6 and tended toward alkaline conditions, the increased concentration of OH^-^ in the solution induced the formation of unstable Fe(OH)_2_, which was further oxidized to Fe(OH)_3_ precipitate, leading to a decrease in chelated iron content.

Ascorbic acid was used as a reducing agent in the reaction system to inhibit the oxidation of ferrous ions [28]. As shown in Figure 1C, its concentration requires precise control. Insufficient concentration leads to the oxidation and precipitation of Fe^2+^, reducing the available substrate for chelation, whereas an excessively high concentration may alter the system’s pH or chemical equilibrium, interfering with the chelation process. The experiment determined that an ascorbic acid concentration of 1.4% provided the best protective effect, resulting in a chelate iron content of 8.90 ± 0.15 g/kg.

The concentration of the oyster peptide substrate significantly influenced the chelation yield. As shown in Figure 1D, this parameter had an optimal threshold. When the peptide concentration was 1%, the chelation effect was optimal, with an iron content as high as 18.53 ± 0.99 g/kg. Beyond this concentration, the iron content decreased instead of increasing. This not only reduced reaction efficiency but also wasted peptide resources. Therefore, 1% was identified as the optimal process concentration.

Finally, Figure 1E reveals the effect of temperature on the reaction [29]. Although the overall influence of temperature on iron content was relatively mild, 35 °C was observed as the optimal reaction temperature. Above 35 °C, the oxidation of ferrous ions accelerated, and peptides might undergo heat-induced side reactions (such as the Maillard reaction), leading to a loss of effective binding sites [30,31]. Below 35 °C, the thermal motion and diffusion rates of molecules were limited, reducing the frequency of effective collisions between substrates. Therefore, controlling the reaction temperature at 35 °C effectively balanced the reaction rate and product stability.

### 3.2. Optimization of Chelation Conditions and Preparation of OP-Fe Chelate

Based on the results of single-factor experiments, the optimal peptide concentration was determined to be 1% and the optimal chelation temperature was set at 35 °C. Three variables peptide-to-iron ratio, ascorbic acid concentration, and pH were selected for optimization using a three-level, three-factor experimental design, with the iron content of the chelate as the response value. The experimental design and corresponding optimization results are presented in Table 1. Under different chelation conditions, the iron content of the chelate ranged from 11.5 g/kg to 17.2 g/kg.

The relationship between the iron content of the chelate (*Y*) and the independent variables is described by the following second-order polynomial model:

$$Y=16.93-0.074A+0.1556B+1.03C+0.27AB-0.646AC+0.2873BC-1.22A^{2}-2B^{2}-2.35C^{2}$$

The analysis of variance (ANOVA), presented in Table 2, shows that the regression model was statistically highly significant (*p* = 0.0002 < 0.01), confirming a strong fit for the data. The influence of the quadratic terms *B^2^* and *C^2^* on the chelate iron content was extremely significant (*p* < 0.01). Significant effects (*p* < 0.05) were also observed for the linear term *C*, the quadratic term *A^2^*, and the interaction term *AC*. All factors studied contributed to the response, though to different extents. Based on the *F*-values, the factors were ranked in descending order of influence on iron content as follows, ascorbic acid concentration (*C*) > pH (*B*) > peptide-to-iron ratio (*A*). The model exhibited a high degree of fit, with a determination coefficient (R^2^) of 0.9887. The adjusted R^2^ (0.9684) and predicted R^2^ (0.9167) further indicated excellent reliability and predictive capability. Adeq Precision value of 20.6592, substantially exceeding the threshold of 4, signifies adequate model discrimination and strong agreement between predicted and experimental values. Therefore, the established quadratic response surface model is both statistically adequate and suitable for the prediction and optimization of oyster peptide iron chelate preparation conditions.

**Figure 2:**
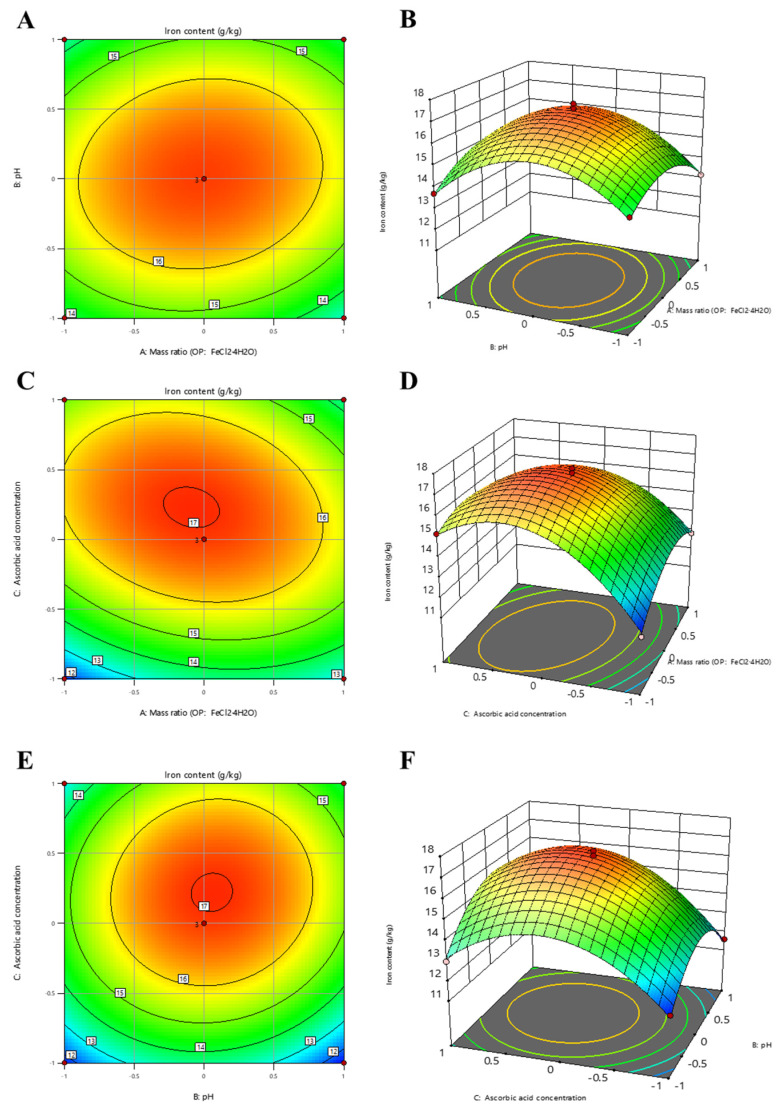
Response surface and contour plots illustrate the effects of interactions among pH, ascorbic acid concentration, and the peptide-to-iron mass ratio on the iron content of the chelate. The surface plots, in particular, display the combined effects on iron content resulting from the interactions of these factors. (**A**) Peptide-to-iron mass ratio and pH. (**C**) Peptide-to-iron mass ratio and ascorbic acid concentration. (**E**) Ascorbic acid concentration and pH. Corresponding contour plots (**B**,**D**,**F**) depict the interactions for (**B**) peptide-to-iron mass ratio and pH. (**D**) Peptide-to-iron mass ratio and ascorbic acid concentration. (**F**) Ascorbic acid concentration and pH.

The interaction between the peptide-to-iron mass ratio and ascorbic acid concentration had a greater effect on the peptide iron chelate than the other two factor pairs, as illustrated by the response surface plots (Figure 2). The iron content of the chelate increased with rising ascorbic acid concentration, before subsequently declining. This decrease may be attributed to excessively high ascorbic acid levels altering the original solution pH, thereby weakening the chelating capacity. Similarly, the iron content increased with changes in the peptide-to-iron mass ratio, but no significant further improvement was observed once the ratio exceeded 3:1. Within the tested range, variations in pH played a positive role, with an appropriate pH value promoting the chelation of ferrous ions by the oyster peptide.

A verification experiment was conducted under the optimized conditions of a peptide-to-iron ratio of 3.91:1, an ascorbic acid concentration of 1.49%, and a pH of 6.05. The resulting iron content of the chelate was 15.44 ± 0.18 g/kg. This value shows good agreement with the model prediction, thereby validating the effectiveness of the established model.

### 3.3. Characterization Results of OP and OP-Fe

#### 3.3.1. FTIR Spectroscopy

**Figure 3:**
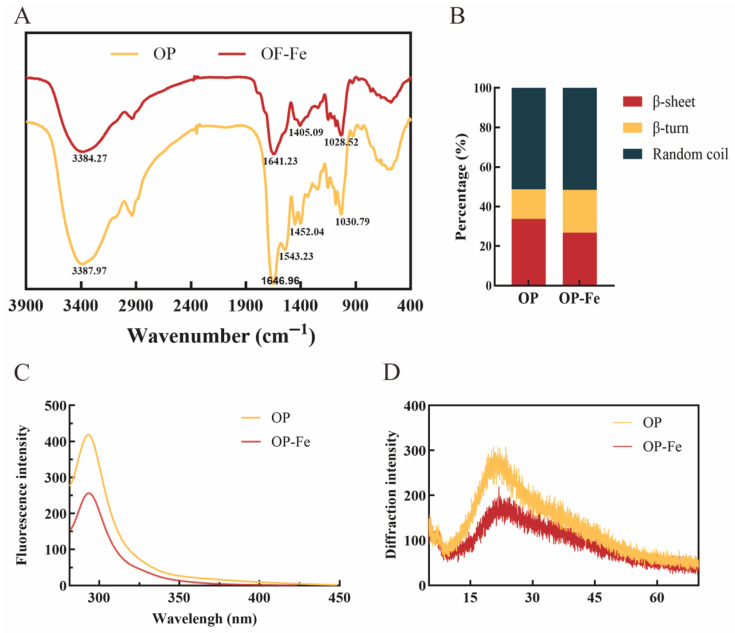
Characterization analysis of OP and OP-Fe. (**A**) Fourier-transform infrared spectroscopy. (**B**) Circular dichroism spectroscopy. (**C**) Fluorescence spectroscopy. (**D**) X-ray diffraction.

In the infrared spectrum (Figure 3A), the absorption peak of the −OH group is typically located between 3600 and 3200 cm^−1^. Based on the FTIR data of oyster peptide and its chelate, the absorption peak of the peptide–iron chelate shifted to approximately 3384 cm^−1^, indicating that the −OH group underwent stretching vibration upon binding with ferrous ions [32]. Following chelation with ferrous iron, the peak originally observed at 1452 cm^−1^ in the oyster-derived peptide was no longer detectable. In standard spectroscopic analysis, absorption bands around 1400 cm^−1^ are commonly associated with the symmetrical stretching vibration of the carboxylate group (−COO^−^) present in amino acid residues [33]. Compared with the raw oyster peptide, the infrared spectrum of the chelate showed significant changes. The absorption peak of the amide I band shifted from 1646 cm^−1^ to 1641 cm^−1^, while the amide II band peak at 1543 cm^−1^ disappeared. These observations suggest that the carbonyl oxygen (C=O) and amide nitrogen (N−H) of the peptide bonds likely participated in the coordination reaction with Fe^2+^ [34].

#### 3.3.2. CD Spectroscopy

Figure 3B displays the percentage composition of secondary structures derived from circular dichroism data. This method is commonly used to investigate structural changes in peptides. The results indicate that the secondary structure of oyster peptide is primarily composed of β-sheets, β-turns, and random coils. From the conformational perspective, the dominance of random coils confers high flexibility and conformational plasticity to the peptide chain. This flexible nature provides a structural basis for Fe^2+^ coordination, allowing the peptide chain to adjust its local spatial structure to accommodate Fe^2+^ binding without requiring extensive conformational rearrangement. According to the literature, metal ions may bind to certain amino acid side chains, leading to twisting or refolding of the peptide chain. The increase in the proportion of β-turns in the chelate may result from the competitive disruption of the hydrogen-bond network that stabilizes β-sheets by Fe^2+^ coordination, while simultaneously inducing local bending of the peptide chain to form β-turn structures that better match the coordination geometry of the metal ion [35,36].

#### 3.3.3. FL Spectroscopy

As shown in Figure 3C, the results indicate that OP and OP-Fe exhibited absorption peaks at 293.2 nm and 293.5 nm, with fluorescence intensities of 418.8 and 256, respectively. This finding demonstrates fluorescence quenching upon the binding of OP with Fe^2+^. The quenching phenomenon may be attributed to the formation of a non-luminescent ground-state complex through the chemical interaction between Fe^2+^ and specific functional groups within the oyster peptide, such as carboxyl (−COOH), amino (−NH_2_), and imidazole groups. Following light absorption, the energy of the excited state of this new complex is dissipated via non-radiative transitions rather than returning to the ground state through fluorescence emission, thereby directly resulting in the observed reduction in fluorescence intensity.

#### 3.3.4. XRD Analysis

XRD is commonly used to determine the crystalline nature of substances, as crystal structures cause incident X-ray beams to diffract at specific angles. The diffractogram (Figure 3D) shows a broad, diffuse diffraction peak between approximately 20° and 25° (2θ). Furthermore, the diffraction intensity of OP-Fe was lower than that of OP, indicating a lack of an ordered, crystalline arrangement in both OP and OP-Fe. Therefore, it can be concluded that OP effectively chelated with Fe^2+^, rather than forming a simple physical mixture [37].

#### 3.3.5. Molecular Docking Analysis

Research indicates that the metal ion-binding capacity of peptides is primarily determined by their amino acid composition. Amino acids such as aspartic acid, glutamic acid, lysine, arginine, and histidine exhibit high affinity for metal ions due to their characteristic side-chain groups, including carboxyl, amino, guanidyl, and imidazolyl groups. Moreover, the metal ion-binding ability of peptides is positively correlated with the content of these amino acids [38,39].

**Figure 4:**
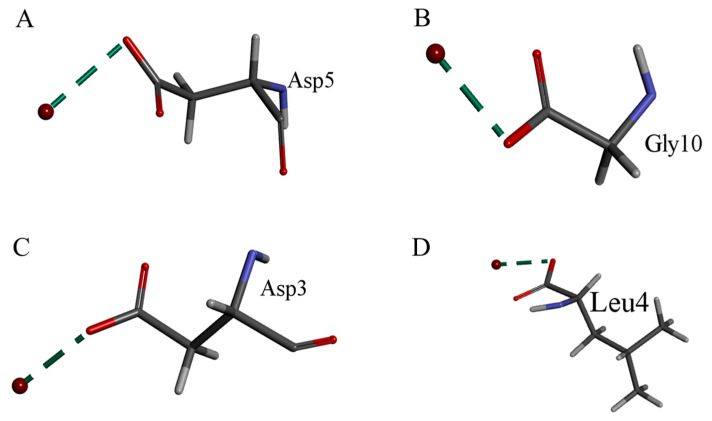
Docking results of amino acid residues from four peptides with ferrous ions, where the formed chemical bonds are indicated by dashed lines. (**A**) Asp5 residue in GDDWDYIPLPRRH with Fe^2+^. (**B**) Gly10 residue in GPM (+15.99) GPPGPPG with Fe^2+^. (**C**) Asp3 residue in LGDDWDYIPLPR with Fe^2+^. (**D**) Leu4 residue in VGPA with Fe^2+^.

Four known peptides were selected for molecular docking analysis, and each was found to possess binding sites for ferrous ions. These sites involve amino acid residues such as Asp5, Gly10, Asp3, and Leu4, respectively (Figure 4).

Based on the results of CD and FTIR. The binding of metal ions disrupts the intermolecular hydrogen-bonding network that stabilizes the β-sheet structure. β-turns, often located on the peptide surface and rich in hydrophilic and flexible residues, frequently serve as binding sites for metal ions. The transition from β-sheet to β-turn is directly reflected by changes in the amide I band. Coordination of metal ions with the carbonyl oxygen (C=O) or amide nitrogen (N) of the peptide backbone leads to shifts in the amide bands. Since Gly is commonly found in turn regions and lacks steric hindrance from side chains, its backbone carbonyl oxygen is likely one of the coordination sites [40]. The side-chain carboxyl groups of Asp are capable of binding with Fe^2+^. This possibility is further supported by the formation of chemical bonds between Fe^2+^ and Asp3 as well as Asp5, which corresponds to the spectral changes observed near 1400 cm^−1^ in the infrared spectroscopy analysis. Moreover, fluorescence spectroscopy revealed that upon the addition of Fe^2+^, the fluorescence emission intensity of the peptide metal chelate significantly decreased compared to that of the free peptide, exhibiting a pronounced fluorescence quenching effect. This phenomenon is likely related to the formation of a static complex, in which the side-chain carboxyl group of Asp and the backbone carbonyl group of Gly act as coordinating atoms to form a stable non-fluorescent ground-state complex with Fe^2+^. Specifically, chemical bonds are formed between Fe^2+^ and Asp3, Asp5, as well as Gly10.

Both Gly and Leu contain amino groups (−NH_2_) in their structures. The formation of chemical bonds between Fe^2+^ and Gly10 as well as Leu4, accompanied by shifts in the absorption peaks around 3400 cm^−1^ in the infrared spectra, indicates that these two amino acids can also bind to Fe^2+^.

### 3.4. Study of Iron Transport Across a Caco-2 Cell Monolayer

#### 3.4.1. Measurement of TEER and Detection of AKP Activity

**Figure 5:**
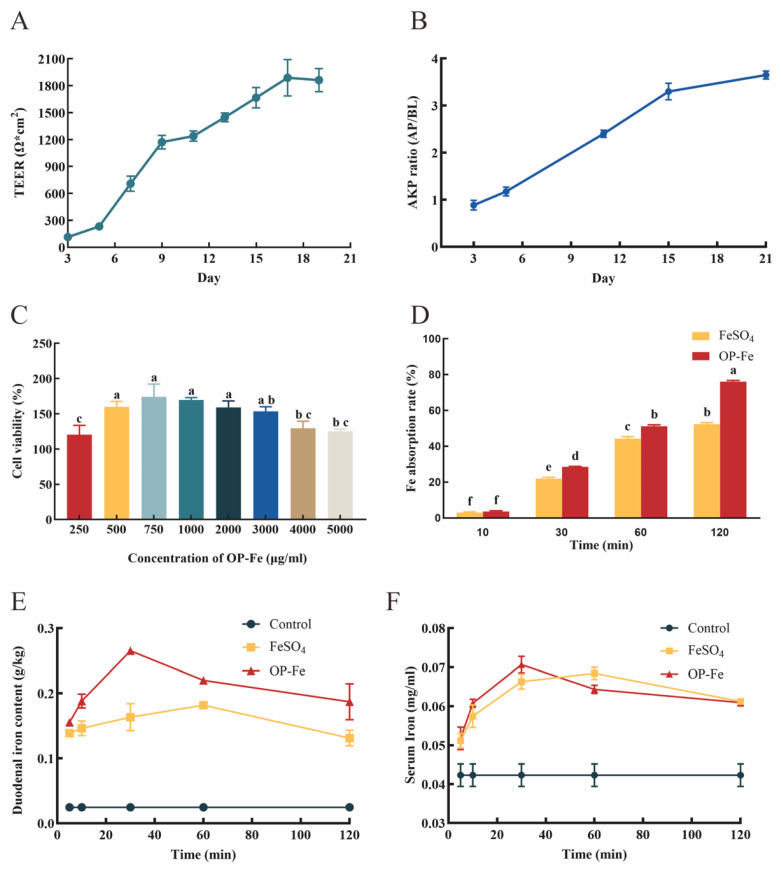
Bioavailability analysis and pharmacokinetics of OP-Fe. (**A**) TEER values of the Caco-2 cell monolayer. (**B**) Relationship between AKP activity and growth time of the Caco-2 cell monolayer. (**C**) Viability of Caco-2 cells. (**D**) Absorption rates of OP-Fe and FeSO_4_ in cells at different time points. (**E**,**F**) Iron content in the small intestine and serum at different time points after administration of OP-Fe and FeSO_4_. The data were analyzed by two-way ANOVA. All data are expressed as average values and standard deviations based on three independent replicates (*n* = 3). Statistically significant differences (*p* < 0.05) among groups are denoted by distinct lowercase superscript letters.

TEER values are positively correlated with the tightness of the cell monolayer. A TEER value exceeding 1000 Ω·cm^2^ is generally considered indicative of a well-formed, tight monolayer suitable for intestinal transport and absorption studies. As shown in Figure 5A, during the initial phase of Caco-2 cell culture, the increase in TEER was relatively gradual. This corresponds mainly to the stages of cell attachment, adaptation, and proliferation on the Transwell polycarbonate membrane, before a confluent monolayer was established. Approximately one week later, after the cells had largely covered the membrane, TEER began to increase exponentially, marking the rapid formation and maturation of intercellular tight junctions. By around day 19, the TEER value had surpassed 1000 Ω·cm^2^, entered a plateau phase, and stabilized, indicating that the cells had fully fused to form an intact and functional barrier structure.

AKP is a marker enzyme localized at the brush border of intestinal epithelial cells. In Caco-2 cell monolayers, its expression serves as an indicator of brush-border formation and, to a certain extent, reflects the degree of cellular differentiation. As shown in Table 3, on day 3 of cell growth, AKP activity on the apical (AP) and basolateral (BL) sides was similar, with the BL side exhibiting slightly higher activity than the AP side. From day 3 to day 11, AP side activity increased steadily. By day 15, AP side AKP activity had risen to 7-fold that of day 3. In contrast, BL side activity increased at a slower rate, showing less than a 2-fold increase by day 15. According to Figure 5B, the AP/BL activity ratio increased from 0.89 ± 0.1 on day 3 to 3.30 ± 0.18 on day 15, indicating that over time, AKP activity became predominantly localized to the AP side. After 21 days of culture, the cells secreted AKP at the apical brush border membrane, confirming that they had acquired the typical functional characteristics of mature intestinal epithelial cells. The monolayer was thus considered suitable for subsequent intestinal transport and absorption experiments [41,42].

#### 3.4.2. Analysis of the Effect of Peptide-Iron Chelate on Caco-2 Cell Absorption

The CCK-8 assay provides a convenient and accurate method for assessing cell proliferation and cytotoxicity. As clearly shown in Figure 5C, the highest cell viability (174.13%) was observed at a sample concentration of 750 µg/mL. Consequently, this concentration was selected for subsequent treatment experiments.

As shown in Figure 5D, the highest Fe^2+^ absorption rate across the Caco-2 cell monolayer was achieved at 120 min, reaching 76.07% for OP-Fe. This value was significantly higher than that of FeSO_4_ (52.39%), indicating that, compared to the inorganic iron salt, OP-Fe can be more effectively digested and absorbed in the small intestine, demonstrating its potential as an effective iron supplement.

#### 3.4.3. Pharmacokinetics

The pharmacokinetic analysis in mice aligned well with the in *vitro* results, showing that iron levels in both serum and the small intestine were higher after administration of OP-Fe than after FeSO_4_. In the small intestine, the iron content of OP-Fe reached 0.27 g/kg at 30 min post administration, while the peak level for FeSO_4_ at 1 h was only 0.18 g/kg (Figure 5E). Similarly, in serum, OP-Fe achieved a peak iron concentration of 0.071 mg/mL at 30 min, whereas FeSO_4_ reached its peak of 0.068 mg/mL at 40 min (Figure 5F).

The elevated levels observed in mice suggest that OP-Fe is absorbed more efficiently in the intestine and is also distributed more effectively throughout the system compared to FeSO_4_ [43]. The stability of the peptide-bound form may prevent the rapid clearance experienced by FeSO_4_, allowing OP-Fe to provide a sustained release of ferrous ions. The peptide ligand likely helps stabilize the ferrous iron, protecting it from rapid degradation or binding in the gastrointestinal environment [44].

### 3.5. Transport Mechanism of OP-Fe Across the Caco-2 Cell Membrane

Currently identified mechanisms for peptide absorption in the small intestine primarily include carrier mediated transport via PepT1, paracellular transport, and transcytosis. Known specific iron transport proteins include DMT1, FPN1, ZIP4, and TFR1 [45].

Ferrous ions (Fe^2+^), after binding with transferrin, form a complex that initiates cellular iron delivery through specific binding to transferrin receptor 1 (TFR1) on the cell surface. This complex is subsequently internalized into the cell via clathrin mediated endocytosis. Within the endosomal compartment, iron is transported across the endosomal membrane into the cytosol by the divalent metal transporter 1 (DMT1). Once in the cytosol, the fate of iron is determined by the cell’s immediate needs: it can be directly transported to sites of utilization for metabolic activities if required, or stored for future use if not immediately needed.

**Figure 6:**
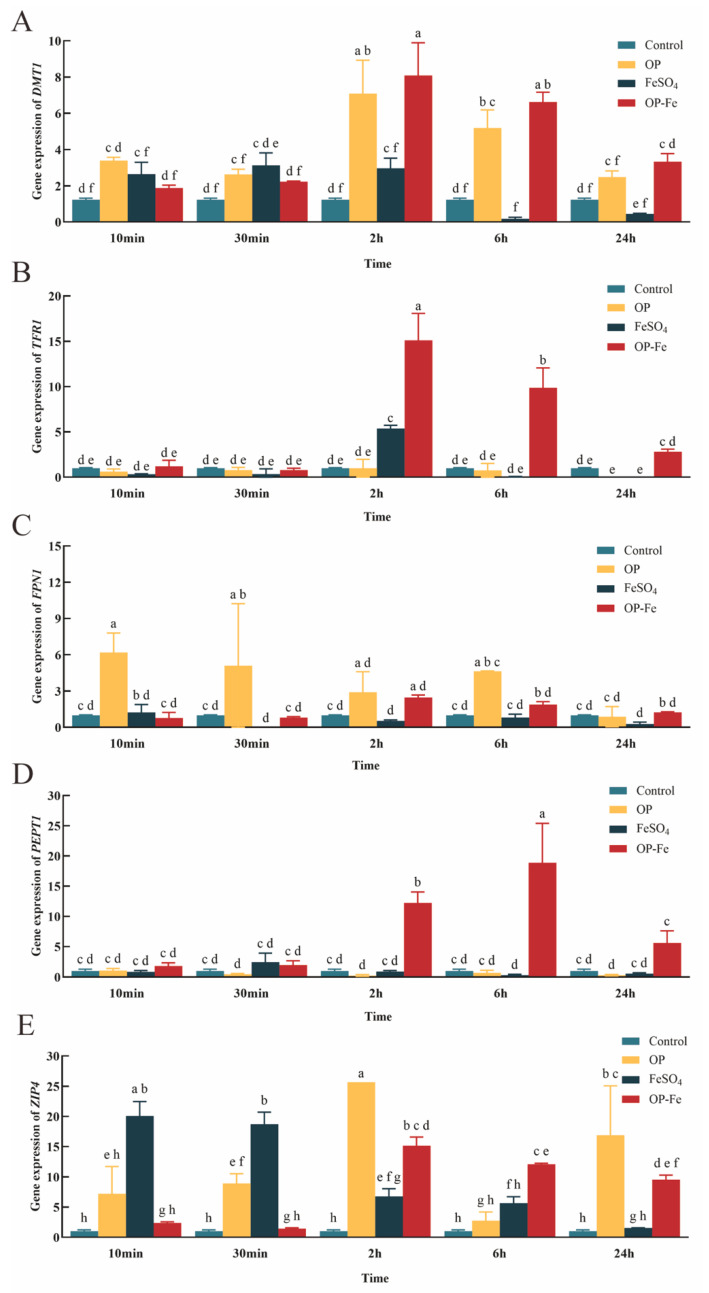
Regulation of different genes by OP, OP-Fe and FeSO_4_. mRNA expression levels of (**A**) DMT1, (**B**) TfR1, (**C**) FPN1, (**D**) PEPT1, and (**E**) ZIP4. The data were analyzed by two-way ANOVA. All values are presented as the mean ± standard deviation (*n* = 3). Different lowercase letters indicate statistically significant differences between groups (*p* < 0.05).

As shown in Figure 6A, as an available iron source, ferrous sulfate (FeSO_4_) is absorbed primarily through the divalent metal transporter 1 (DMT1) located on the apical membrane of small intestinal epithelial cells. When a large amount of free Fe^2+^ enters the cells within a short period, intracellular iron levels rise rapidly. To maintain iron homeostasis, the cells activate a post transcriptional negative feedback mechanism, leading to significant suppression of DMT1 expression after 6 h [46]. In contrast, the OP-Fe treatment group displayed a distinct pattern. The peptide ligand may act as a buffer or carrier, releasing iron into the intracellular iron pool in a more controlled manner, thereby preventing a sharp surge in cytoplasmic iron concentration. Since this does not trigger a strong iron-overload signal, the cellular perception of sufficient iron is relatively mild. As a result, the suppression of DMT1 transcription is delayed and more gradual. The peak expression observed at 2 h suggests that cells still require iron at the initial stage and that OP-Fe can effectively meet this demand. The subsequent slow decline reflects the gradual engagement of homeostatic regulation as iron supply continues. Collectively, these results indicate that compared with FeSO_4_ treatment, OP-Fe can supply iron to cells more efficiently and maintain higher DMT1 expression levels over a longer period. The FeSO_4_ treatment group began to significantly upregulate TFR1 expression at 2 h, followed by a rapid return to basal levels at 6 h and 24 h (Figure 6B). This response occurs because TFR1 expression is tightly downregulated by intracellular iron levels. When free iron rapidly enters the cells, the binding activity of iron-regulatory proteins (IRPs) to iron-responsive elements (IREs) decreases, leading to accelerated degradation of TFR1 mRNA. The transient upregulation observed at 2 h in the FeSO_4_ group likely reflects a dynamic adjustment during the initial iron influx; the subsequent rapid decline indicates that the free iron quickly activates negative feedback, shifting the cells into an iron-sufficient state. In contrast, the OP-Fe treatment group showed the strongest induction of TFR1 expression at 2 h, far exceeding the FeSO_4_ group. Although its expression declined at 6 h and 24 h, its level at 24 h remained higher than all other groups. This pattern can be attributed to the delayed release of chelated iron inside the cells, which keeps the IRPs in a state of low-iron perception. By sustaining high TFR1 expression, the cells continuously enhance the transferrin-mediated endocytic cycle, establishing a positive loop of iron absorption, utilization, and re-uptake, thereby avoiding the feedback-driven shutdown of absorption induced by FeSO_4_. The OP-Fe complex significantly increases cellular iron demand and accelerates iron uptake. Therefore, OP-Fe not only acts as an iron carrier but also achieves a metabolically adapted iron supply by regulating core components of iron homeostasis, which represents its fundamental advantage over inorganic iron.

FPN1 is currently the only known transporter protein capable of exporting iron ions from inside to outside the cell [47]. As shown in Figure 6C, the FPN1 expression level in the OP only treatment group remained consistently higher than that in the Control group throughout the experimental period. Acting as a chelator, OP may bind to a portion of intracellular Fe^2+^, creating a cellular perception of functional iron deficiency. This could relieve the suppression of FPN1 transcription and promote iron export in an attempt to restore homeostasis. The biphasic peaks observed at 10 min and 6 h may correspond to rapid signaling activation and subsequent adaptive transcriptional regulation, respectively. In the FeSO_4_ treatment group, FPN1 expression was comparable to or only slightly higher than the Control at all time points, without the expected significant suppression. This suggests that under these experimental conditions, FPN1 transcription was relatively insensitive to iron concentration, and the activity of the FPN1 protein was likely governed primarily by post translational regulation specifically, hepcidin mediated degradation rather than transcriptional downregulation. The OP-Fe group showed expression levels similar to or slightly higher than the Control at all time points, with relatively stable expression throughout the process and a moderately stronger induction than the FeSO_4_ group. This may be because OP-Fe supplies iron in a chelated form, avoiding a sharp rise in intracellular free-iron concentration and thereby not triggering a strong signal for FPN1 transcriptional suppression. By buffering the acute fluctuations in iron signaling through chelation, OP-Fe helps maintain a more balanced and controlled expression of core iron-homeostasis transporters such as FPN1. This mechanism may contribute to a safer and more sustained iron supply, providing deeper mechanistic support for its potential as an advantageous iron supplement.

SLC15A1 (PepT1) is primarily localized to the apical membrane of intestinal epithelial cells (duodenum, jejunum, and ileum) and serves as the major mediator for dietary peptide absorption [41,48]. Figure 6D shows changes in PEPT1 gene expression under four different treatments across various time points. PepT1 is primarily responsible for transporting di- and tri-peptides from the intestinal lumen into enterocytes and serves as a key protein for nutrient absorption. FeSO_4_ exerted a very minor effect on PepT1 expression at all time points, with expression levels almost identical to those in the Control group, indicating its negligible role in the transcriptional regulation of PepT1. In contrast, OP-Fe, as a peptide iron complex, exhibits an overall structure more closely resembling that of a peptide. Cells may recognize and absorb it via the peptide transporter PepT1. Since PepT1 mediated uptake internalizes the entire complex, the iron ion chelated by the peptide ligand may not be immediately or completely released as free iron upon entering the cytosol. This results in a more gradual or delayed increase in intracellular free iron concentration. Consequently, the cellular perception of an iron overload signal is weaker or delayed, which explains why the strong negative feedback suppression of DMT1 does not occur immediately with OP-Fe treatment, despite efficient iron absorption. Meanwhile, to meet metabolic demands, cells may upregulate PepT1, which is responsible for taking up this complex, leading to a significant increase in its expression within 2–6 h. This observation suggests that OP-Fe achieves a regulated or gentler form of iron delivery via the PepT1 pathway. On one hand, it leverages the high efficiency of the peptide transport system; on the other hand, its slow-release characteristics likely mitigate the sudden surge in iron concentration that could disrupt cellular homeostasis, thereby avoiding a strong negative feedback response. Therefore, compared to FeSO_4_, which directly impacts the DMT1 mediated iron homeostasis system, the PepT1 dependent absorption mechanism of OP-Fe may enable cells to maintain a higher, yet more controlled, iron-uptake capacity over an extended period [48].

ZIP4 belongs to the ZIP family, whose members are primarily responsible for transporting metal ions into cells. Existing experiments have confirmed that iron and zinc mutually inhibit each other’s absorption [49,50]. Given that ZIP4 has a dual function in transporting both iron and zinc in hepatocytes [51], this transporter likely plays a key mediating role in their interaction. As shown in Figure 6E, the induction of ZIP4 expression by OP is consistent with expectations, as ZIP4 is a major zinc-uptake transporter typically upregulated during zinc deficiency to compensate for the lack. However, the observation that OP-Fe also induced ZIP4 expression in the short term reveals a complex interaction between zinc and iron in cellular homeostasis, where competition exists between the two metals during intestinal absorption and intracellular transport. In OP-Fe, Fe^2+^ enters the cell in a chelated form, which may slow the release of free iron. As a result, the cellular perception of zinc deficiency is not immediately overridden, allowing transient upregulation of ZIP4 to still occur [52]. In contrast, traditional inorganic iron such as FeSO_4_ often rapidly elevates free-iron levels and strongly inhibits zinc absorption. Through the buffering effect of the peptide ligand, OP-Fe may mitigate the disruption of zinc homeostasis and help maintain a balance between iron and zinc absorption. This characteristic is particularly important in long-term iron-supplementation applications, as it could reduce the risk of zinc deficiency induced by excessive iron intake.

## 4. Conclusions

This study determined the best conditions for combining oyster peptides with ferrous ions using single-factor tests and response surface methods. Analysis of the structure and molecular docking showed that ferrous ions bind to groups like –COOH and –NH_2_ in the peptides. This creates a new type of chelate that is stable but can also flex in shape. Tests with Caco-2 cells and animal studies showed that OP-Fe is better than traditional ferrous sulfate (FeSO_4_) at being absorbed by the gut and is processed differently in the body. This likely happens because OP-Fe increases the activity of important transport genes like PEPT1 and TFR1 in cells. OP-Fe is efficiently taken up by cells through specific transport pathways. Its chelate structure acts like a buffer, releasing iron slowly inside cells. This prevents a sudden jump in free iron that would normally trigger strong negative feedback, allowing iron absorption to stay steady over time. These results show that OP-Fe effectively helps living organisms absorb and use Fe^2+^. It has the potential to be developed into a new, efficient iron supplement that causes less irritation.

## Figures and Tables

**Table 1:** Box–Behnken Design and Results for Optimization of Chelation Process.

Run	Peptide-to-Iron Ratio	pH	Ascorbic Acid Concentration (%)	Iron Content of the Chelate
1	3:1	4	1.8	12.99
2	5:1	6	0.2	12.833
3	3:1	6	1	17.037
4	1:1	4	1	13.986
5	5:1	4	1	13.152
6	3:1	8	1.8	13.93
7	5:1	8	1	13.949
8	1:1	6	1.8	15.162
9	3:1	6	1	16.473
10	3:1	6	1	17.268
11	5:1	6	1.8	13.868
12	3:1	8	0.2	11.578
13	3:1	4	0.2	11.787
14	1:1	8	1	13.703
15	1:1	6	0.2	11.543

**Table 2:** ANOVA for the response surface quadratic model.

Source	Sum of Squares	df	Mean Square	F-Value	*p*-Value
Module	46.81	9	5.20	48.7	0.0002
*A*	0.0438	1	0.0438	0.4102	0.5501
*B*	0.1938	1	0.1938	1.81	0.2358
*C*	8.42	1	8.42	78.87	0.0003
*AB*	0.2916	1	0.2916	2.73	0.1594
*AC*	1.67	1	1.67	15.63	0.0108
*BC*	0.3301	1	0.3301	3.09	0.1391
*A^2^*	5.53	1	5.53	51.81	0.0008
*B^2^*	14.83	1	14.38	138.90	<0.0001
*C^2^*	20.40	1	20.40	190.99	<0.0001
Residual	0.5340	5	0.1068		
Lack of Fit	0.1995	3	0.0665	0.3976	0.7717
Pure Error	0.3345	2	0.1672		
Cor Total	47.35	14			

R^2^ = 0.9887. R^2^ADJ = 0.9687. R^2^PRE = 0.9167. Adeg Precision = 20.6592.

**Table 3:** Relationship between AKP Activity and Culture Time in Caco-2 Cell Monolayers (*n* = 3).

Day	AP	BL	AP/BL
3	0.35 ± 0.06 ^e^	0.39 ± 0.03 ^d^	0.89 ± 0.1 ^e^
5	0.53 ± 0.02 ^d^	0.45 ± 0.02 ^b^	1.18 ± 0.09 ^d^
11	1.61 ± 0.02 ^c^	0.69 ± 0.02 ^b^	2.35 ± 0.1 ^c^
15	2.39 ± 0.21 ^b^	0.73 ± 0.07 ^b^	3.30 ± 0.18 ^b^
21	3.41 ± 0.02 ^a^	0.93 ± 0.02 ^a^	3.64 ± 0.08 ^a^

Different lowercase letters indicate significant differences (*p* < 0.05).

## Data Availability

The original contributions presented in this study are included in the article. Further inquiries can be directed to the corresponding authors.

## Supplementary Materials

The following supporting information can be downloaded at: https://www.mdpi.com/article/10.3390/foods15020362/s1, Table S1: Detailed peptide information (including peptide sequence, hydrophobicity (−10lgP), molecular weight, length, ppm, *m*/*z*, retention time (RT), peak area, fraction, scan number, source file, feature count, and identification method.
