# Supplementary material for: Oyster Peptide-Ferrous Chelate Preparation Optimization Structural Characteristics and Enhanced Bioavailability
Source: Foods. 2026 Jan 20;15(2):362. doi: 10.3390/foods15020362 (PMC12840548; doi:10.3390/foods15020362)
Supplement: Supplementary file 1 [file foods-15-00362-s001.zip › foods-4089730-supplementary.pdf]

**Table S1.** Peptide information

| Peptide | -10lgP | Mass | Length | ppm | m/z | RT | Area 1 | Fraction | Scan | Source File | #Feature | Found By |
|---|---|---|---|---|---|---|---|---|---|---|---|---|
| GPQ(+.98)GPSGEPGPEGPAGPIGP | 20.76 | 1754.811 | 20 | 4.5 | 439.712 | 0.56 | 1.3179E6 | 1 | 444 | YS_1762.raw | 1 | PEAKS DB |
| PQGPQ(+.98)NVGFHPPQQFPNQR | 27.63 | 2173.0452 | 19 | -5.5 | 1087.5239 | 14.85 | 1.589E6 | 1 | 7257 | YS_1762.raw | 1 | PEAKS DB |
| GPSGEPGPEGPAGPIGPR | 47.67 | 1627.7954 | 18 | 0.7 | 814.9055 | 14.98 | 1.4513E6 | 1 | 7304 | YS_1762.raw | 1 | PEAKS DB |
| EGPPGTKGDAGLEGAP | 26.56 | 1451.6892 | 16 | 1.1 | 726.8527 | 18.47 | 1.4527E6 | 1 | 8985 | YS_1762.raw | 1 | PEAKS DB |
| GTPPTGKRSNGHAN(+.98)IG | 21 | 1563.7754 | 16 | -7.9 | 782.8888 | 9.24 | 6.103E6 | 1 | 4499 | YS_1762.raw | 1 | PEAKS DB |
| GEPGPEGPAGPIGPR | 45.14 | 1386.6891 | 15 | 0.5 | 694.3522 | 13.77 | 2.8957E6 | 1 | 6712 | YS_1762.raw | 1 | PEAKS DB |
| DAPRAVFPSIVGRPR | 40.75 | 1636.9161 | 15 | 0.2 | 546.6461 | 21.99 | 4.1087E5 | 1 | 10720 | YS_1762.raw | 1 | PEAKS DB |
| HNEHAPNHDNPGDL | 38.2 | 1565.6606 | 14 | -1.2 | 522.8936 | 3.93 | 2.0385E6 | 1 | 1793 | YS_1762.raw | 1 | PEAKS DB |
| VGPFDPQDPDFQY | 37.08 | 1523.6569 | 13 | 1.7 | 762.837 | 45.66 | 1.0973E5 | 1 | 21867 | YS_1762.raw | 1 | PEAKS DB |
| PQGPQNVGFHPPQ | 21.08 | 1401.679 | 13 | 8.2 | 701.8525 | 17.33 | 1.7249E5 | 1 | 8496 | YS_1762.raw | 1 | PEAKS DB |
| LTDDQVDEIIRNT | 43.29 | 1530.7526 | 13 | 0.9 | 766.3843 | 35.07 | 2.4383E5 | 1 | 16923 | YS_1762.raw | 1 | PEAKS DB |
| GDNEPSQAPAGPL | 22.25 | 1251.5731 | 13 | -0.2 | 626.7937 | 24.26 | 3.5244E6 | 1 | 11796 | YS_1762.raw | 1 | PEAKS DB |
| GLPGLPGPVGPPG | 24.3 | 1113.6182 | 13 | 9.8 | 557.8218 | 18.69 | 3.6536E6 | 1 | 9103 | YS_1762.raw | 1 | PEAKS DB |
| NSPPPVPSSQGTP | 22.41 | 1263.6095 | 13 | -0.6 | 632.8116 | 16.23 | 8.0692E6 | 1 | 7842 | YS_1762.raw | 1 | PEAKS DB |
| PQGPPGQPGSPG | 20.48 | 1074.5094 | 12 | 0.5 | 538.2623 | 9.94 | 1.0919E7 | 1 | 4816 | YS_1762.raw | 1 | PEAKS DB |
| PGLPGPQGMPGP | 20.07 | 1103.5433 | 12 | -8.4 | 552.7743 | 47.32 | 1.1162E5 | 1 | 22697 | YS_1762.raw | 1 | PEAKS DB |
| RAPVPLPDVIEE | 33.23 | 1333.7241 | 12 | 1.7 | 667.8705 | 39.69 | 1.1704E5 | 1 | 19079 | YS_1762.raw | 1 | PEAKS DB |
| LGDDWDYIPLPR | 52.8 | 1458.7142 | 12 | 1.3 | 730.3654 | 49.69 | 1.2265E5 | 1 | 23746 | YS_1762.raw | 1 | PEAKS DB |
| GLPGTPGQPGLP | 26.92 | 1089.5818 | 12 | 0.5 | 545.7985 | 10.71 | 2.2936E6 | 1 | 5254 | YS_1762.raw | 1 | PEAKS DB |
| GLDGLNGPPGSP | 28.64 | 1079.5247 | 12 | -5.6 | 540.7666 | 7.59 | 2.6154E6 | 1 | 3695 | YS_1762.raw | 1 | PEAKS DB |
| DVDLKPVPIEVL | 22.77 | 1335.7649 | 12 | 0.8 | 668.8903 | 51.91 | 3.0031E4 | 1 | 24778 | YS_1762.raw | 1 | PEAKS DB |
| VGPFDPQDPDFQ | 26.69 | 1360.5935 | 12 | -0.3 | 681.3038 | 38.85 | 3.3807E5 | 1 | 18611 | YS_1762.raw | 1 | PEAKS DB |
| LTDDQVDEIIRN | 52.25 | 1429.7048 | 12 | -2.5 | 715.8579 | 26.06 | 4.8411E5 | 1 | 12654 | YS_1762.raw | 1 | PEAKS DB |
| IGPTGPPGPPGR | 21.38 | 1101.593 | 12 | -0.1 | 551.8037 | 12.43 | 5.2621E6 | 1 | 6068 | YS_1762.raw | 1 | PEAKS DB |

**Table S1 continued.** Peptide information

| Peptide | -10lgP | Mass | Length | ppm | m/z | RT | Area 1 | Fraction | Scan | Source File | #Feature | Found By |
|---|---|---|---|---|---|---|---|---|---|---|---|---|
| GGEGPPGAPGTP | 30.52 | 992.4563 | 12 | -1.4 | 993.4622 | 10.59 | 6.0097E6 | 1 | 5165 | YS_1762.raw | 1 | PEAKS DB |
| LTDDQVDEIIR | 45.47 | 1315.6619 | 11 | 1.5 | 658.8392 | 26.52 | 1.0523E6 | 1 | 12857 | YS_1762.raw | 1 | PEAKS DB |
| AVFPSIVGRPR | 41.06 | 1197.6981 | 11 | -0.6 | 400.2397 | 18.83 | 2.4143E6 | 1 | 9116 | YS_1762.raw | 1 | PEAKS DB |
| PGEAGPTGAPG | 21.68 | 909.4192 | 11 | -2 | 910.4246 | 4.63 | 2.4252E7 | 1 | 1676 | YS_1762.raw | 1 | PEAKS DB |
| KGNIGDTGPAG | 21.68 | 985.4828 | 11 | 7.4 | 986.4974 | 28.63 | 2.9751E7 | 1 | 13678 | YS_1762.raw | 1 | PEAKS DB |
| PGPPGM(+15.99)RGPPG | 23.84 | 1034.4967 | 11 | -0.9 | 518.2552 | 7.59 | 3.5287E8 | 1 | 3544 | YS_1762.raw | 1 | PEAKS DB |
| FGPAGLM(+15.99)GAGP | 20.37 | 989.464 | 11 | -8.4 | 495.7351 | 7.28 | 4.9627E6 | 1 | 3566 | YS_1762.raw | 1 | PEAKS DB |
| PGPVGVKVHGP | 26.76 | 1042.5923 | 11 | -0.5 | 522.3032 | 15.91 | 5.1068E6 | 1 | 7733 | YS_1762.raw | 1 | PEAKS DB |
| GPAGPLGPPGE | 36.11 | 947.4712 | 11 | -0.2 | 474.7428 | 17.52 | 5.9727E6 | 1 | 8520 | YS_1762.raw | 1 | PEAKS DB |
| REYPEIDPIIL | 31.14 | 1356.7289 | 11 | 0.9 | 679.3723 | 48.57 | 6.3656E4 | 1 | 23213 | YS_1762.raw | 1 | PEAKS DB |
| GVPPPGIPGIP | 26.32 | 999.5753 | 11 | 1.6 | 500.7957 | 43.42 | 9.1397E5 | 1 | 20772 | YS_1762.raw | 1 | PEAKS DB |
| GDDWDYIPLPR | 55.1 | 1345.6302 | 11 | 0.3 | 673.8226 | 46.07 | 9.8452E5 | 1 | 22000 | YS_1762.raw | 1 | PEAKS DB |
| VAAPQPQ(+.98)PAA | 29.4 | 949.4869 | 10 | -1.2 | 950.493 | 11.79 | 1.1119E6 | 1 | 5775 | YS_1762.raw | 1 | PEAKS DB |
| PGEAGPTGAP | 28.97 | 852.3977 | 10 | 0.1 | 853.405 | 0.01 | 1.3462E8 | 1 | 1887 | YS_1762.raw | 1 | PEAKS DB |
| GPSGIRGEPG | 32.2 | 925.4617 | 10 | 0.2 | 463.7382 | 4.52 | 1.578E7 | 1 | 2133 | YS_1762.raw | 1 | PEAKS DB |
| GPM(+15.99)GPPGPPG | 31.77 | 878.3956 | 10 | -0.4 | 879.4026 | 10.97 | 1.6021E7 | 1 | 5331 | YS_1762.raw | 1 | PEAKS DB |
| EGPAGPIGPR | 29.34 | 949.498 | 10 | 0.4 | 475.7565 | 8.3 | 1.9834E7 | 1 | 4001 | YS_1762.raw | 3 | PEAKS DB |
| DDWDYIPLPR | 48.98 | 1288.6088 | 10 | 0.9 | 645.3123 | 48.02 | 2.2827E5 | 1 | 22952 | YS_1762.raw | 1 | PEAKS DB |
| IIAPPERKYS | 25.67 | 1172.6553 | 10 | -0.4 | 391.8922 | 8.61 | 2.3653E6 | 1 | 4203 | YS_1762.raw | 1 | PEAKS DB |
| DDQVDEIIRN | 41.72 | 1215.5731 | 10 | -0.4 | 608.7936 | 21.35 | 2.7507E5 | 1 | 10391 | YS_1762.raw | 1 | PEAKS DB |
| GFAGDDAPRA | 32.88 | 975.441 | 10 | 1 | 488.7283 | 8.96 | 2.9184E6 | 1 | 4344 | YS_1762.raw | 1 | PEAKS DB |
| PGAPGEPGPQ | 27.16 | 905.4243 | 10 | -1.2 | 906.4305 | 6 | 2.9661E8 | 1 | 2742 | YS_1762.raw | 2 | PEAKS DB |
| VRLLLPGELA | 36.06 | 1079.6703 | 10 | 0.7 | 540.8428 | 43.29 | 4.0174E5 | 1 | 20726 | YS_1762.raw | 1 | PEAKS DB |
| QPGM(+15.99)VAPQPG | 20.25 | 996.4698 | 10 | -5 | 997.4722 | 18.37 | 4.8767E6 | 1 | 8903 | YS_1762.raw | 1 | PEAKS DB |

**Table S1 continued.** Peptide information

| Peptide | -10lgP | Mass | Length | ppm | m/z | RT | Area 1 | Fraction | Scan | Source File | #Feature | Found By |
|---|---|---|---|---|---|---|---|---|---|---|---|---|
| GVAGPQGLPG | 23.2 | 851.4501 | 10 | 0.2 | 852.4575 | 22.82 | 5.0926E6 | 1 | 11050 | YS_1762.raw | 1 | PEAKS DB |
| LQGPIGFLEF | 30.01 | 1119.5964 | 10 | 6.8 | 560.8093 | 12.5 | 5.2693E6 | 1 | 6093 | YS_1762.raw | 1 | PEAKS DB |
| IIKDLPIDDF | 33.72 | 1187.6438 | 10 | -0.5 | 594.8289 | 43.89 | 5.515E4 | 1 | 21022 | YS_1762.raw | 1 | PEAKS DB |
| GPIGPEGPPG | 24.47 | 876.4341 | 10 | 0.7 | 439.2246 | 1.23 | 8.9394E6 | 1 | 514 | YS_1762.raw | 1 | PEAKS DB |
| VSLPGPMMGP | 25.05 | 984.4772 | 10 | 0.7 | 985.4852 | 39.79 | 9.1194E4 | 1 | 19114 | YS_1762.raw | 1 | PEAKS DB |
| QGIPSGPGAP | 25.49 | 879.445 | 10 | -0.5 | 880.4518 | 11.79 | 9.7722E6 | 1 | 5741 | YS_1762.raw | 1 | PEAKS DB |
| GPAGPIGPR | 35.45 | 820.4555 | 9 | 0.5 | 411.2352 | 5.65 | 1.1449E7 | 1 | 2715 | YS_1762.raw | 1 | PEAKS DB |
| PSGPPQGTP | 23.3 | 836.4028 | 9 | -1.4 | 837.4089 | 6.86 | 1.1557E7 | 1 | 3316 | YS_1762.raw | 1 | PEAKS DB |
| DEHIPESPF | 30.13 | 1069.4716 | 9 | 0.2 | 535.7432 | 28.37 | 1.203E6 | 1 | 13713 | YS_1762.raw | 1 | PEAKS DB |
| PSQAPAGPL | 21.54 | 836.4392 | 9 | -0.2 | 837.4463 | 16.1 | 1.4219E8 | 1 | 7808 | YS_1762.raw | 1 | PEAKS DB |
| LQGPPGPSG | 23.3 | 808.4079 | 9 | -1.2 | 809.4142 | 11.82 | 1.6599E6 | 1 | 5732 | YS_1762.raw | 1 | PEAKS DB |
| GATGPPGAT | 20.41 | 727.35 | 9 | -2.9 | 728.3552 | 4.33 | 1.7091E6 | 1 | 2023 | YS_1762.raw | 1 | PEAKS DB |
| VRLLLPGEL | 24.76 | 1008.6331 | 9 | 0.4 | 505.324 | 44.83 | 1.7966E5 | 1 | 21451 | YS_1762.raw | 1 | PEAKS DB |
| DWDYIPLPR | 33.37 | 1173.5818 | 9 | 1.3 | 587.799 | 47.26 | 2.2758E5 | 1 | 22630 | YS_1762.raw | 1 | PEAKS DB |
| SGPPQGTPQ | 26.42 | 867.4086 | 9 | 0 | 868.4159 | 0.93 | 2.2821E6 | 1 | 461 | YS_1762.raw | 1 | PEAKS DB |
| GIPSGPGAP | 25.29 | 751.3864 | 9 | -0.5 | 752.3933 | 11.15 | 2.5093E8 | 1 | 5385 | YS_1762.raw | 2 | PEAKS DB |
| PGEPGPMGP | 28 | 837.3691 | 9 | -0.5 | 838.376 | 12.3 | 2.5734E7 | 1 | 5987 | YS_1762.raw | 2 | PEAKS DB |
| KPDRPAGAP | 26.22 | 907.4875 | 9 | -1 | 454.7505 | 4.33 | 3.6947E6 | 1 | 2052 | YS_1762.raw | 1 | PEAKS DB |
| GLQ(+.98)GPIGLP | 21.29 | 851.4752 | 9 | -0.2 | 852.4824 | 43.75 | 3.719E5 | 1 | 20932 | YS_1762.raw | 1 | PEAKS DB |
| DEHVPGSPF | 25.18 | 983.4348 | 9 | 1.3 | 492.7253 | 22.28 | 3.9433E5 | 1 | 10843 | YS_1762.raw | 1 | PEAKS DB |
| IIAPPERKY | 27.94 | 1085.6233 | 9 | 0.2 | 362.8818 | 8.86 | 4.0875E6 | 1 | 4312 | YS_1762.raw | 1 | PEAKS DB |
| GPQGPIGPR | 41.14 | 877.4769 | 9 | 0 | 439.7457 | 3.23 | 4.1642E8 | 1 | 1592 | YS_1762.raw | 2 | PEAKS DB |
| GPSGPPGPA | 38.74 | 735.3551 | 9 | 0.4 | 736.3627 | 46.88 | 4.4631E5 | 1 | 23358 | YS_1762.raw | 2 | PEAKS DB |
| SGPPGPAGP | 21.02 | 735.3551 | 9 | -3.3 | 736.36 | 44.22 | 5.4536E4 | 1 | 21261 | YS_1762.raw | 1 | PEAKS DB |

**Table S1 continued.** Peptide information

| Peptide | -10lgP | Mass | Length | ppm | m/z | RT | Area 1 | Fraction | Scan | Source File | #Feature | Found By |
|---|---|---|---|---|---|---|---|---|---|---|---|---|
| GPSPAGPSP | 21.19 | 765.3657 | 9 | -6.4 | 766.368 | 8.23 | 5.7126E6 | 1 | 4089 | YS_1762.raw | 1 | PEAKS DB |
| GFAGDDAPR | 36.97 | 904.4039 | 9 | -0.4 | 453.209 | 4.7 | 8.2826E6 | 1 | 2249 | YS_1762.raw | 1 | PEAKS DB |
| GPIGPPGPS | 33.72 | 777.402 | 9 | -1.5 | 778.4081 | 11.47 | 8.3304E6 | 1 | 5557 | YS_1762.raw | 1 | PEAKS DB |
| PQQPEAAPQ | 31.78 | 964.4614 | 9 | -0.1 | 965.4685 | 8.23 | 8.5326E6 | 1 | 3976 | YS_1762.raw | 1 | PEAKS DB |
| PVGPPGLP | 24.57 | 732.417 | 8 | 0.2 | 733.4244 | 23.14 | 1.0132E6 | 1 | 11257 | YS_1762.raw | 1 | PEAKS DB |
| IGIGISGQ | 33.43 | 743.4177 | 8 | -0.4 | 744.4247 | 29.46 | 1.1232E7 | 1 | 14217 | YS_1762.raw | 1 | PEAKS DB |
| PQGPIGPR | 31.37 | 820.4555 | 8 | -1.1 | 411.2346 | 6.73 | 1.2246E7 | 1 | 3228 | YS_1762.raw | 1 | PEAKS DB |
| QPEAPQPA | 22.28 | 836.4028 | 8 | -0.8 | 837.4094 | 5.62 | 1.6161E8 | 1 | 2870 | YS_1762.raw | 1 | PEAKS DB |
| PQGPPGTP | 36.89 | 749.3708 | 8 | -0.8 | 750.3774 | 5.87 | 1.8353E7 | 1 | 2816 | YS_1762.raw | 1 | PEAKS DB |
| EGATGLTQ | 30.25 | 775.3712 | 8 | -1 | 776.3777 | 5.93 | 1.9839E7 | 1 | 2852 | YS_1762.raw | 1 | PEAKS DB |
| VGPVGPSG | 25.02 | 668.3493 | 8 | -0.2 | 669.3564 | 8.04 | 2.8972E6 | 1 | 3925 | YS_1762.raw | 1 | PEAKS DB |
| NPAPNPPQ | 25.39 | 833.4031 | 8 | -1 | 834.4095 | 7.97 | 3.452E6 | 1 | 3889 | YS_1762.raw | 1 | PEAKS DB |
| TIPIDGDL | 25.19 | 842.4385 | 8 | -0.2 | 843.4457 | 33.31 | 3.4756E5 | 1 | 16101 | YS_1762.raw | 1 | PEAKS DB |
| PQQPEAAP | 29.72 | 836.4028 | 8 | 1 | 837.4109 | 5.11 | 3.6833E8 | 1 | 2604 | YS_1762.raw | 1 | PEAKS DB |
| AVFPSIVG | 37.27 | 788.4432 | 8 | -0.2 | 789.4504 | 41.67 | 3.7982E5 | 1 | 19984 | YS_1762.raw | 1 | PEAKS DB |
| SPGPVGPQ | 20.41 | 737.3708 | 8 | -0.5 | 738.3777 | 7.28 | 4.9612E7 | 1 | 3639 | YS_1762.raw | 1 | PEAKS DB |
| APGPVGRP | 26.24 | 749.4184 | 8 | 1.4 | 375.717 | 0.95 | 5.2579E5 | 1 | 244 | YS_1762.raw | 1 | PEAKS DB |
| TSCCSGPK | 24.05 | 781.3099 | 8 | 5.9 | 391.6645 | 50.02 | 5.7548E3 | 1 | 23892 | YS_1762.raw | 1 | PEAKS DB |
| QPGPGVPV | 22.49 | 749.4072 | 8 | -0.8 | 750.4138 | 14.1 | 6.6159E6 | 1 | 6852 | YS_1762.raw | 1 | PEAKS DB |
| GPFGPAGL | 28.5 | 714.3701 | 8 | 5.3 | 715.3811 | 30.91 | 8.0513E5 | 1 | 14942 | YS_1762.raw | 1 | PEAKS DB |
| IAPPERKY | 29.64 | 972.5392 | 8 | 0.1 | 325.187 | 4.98 | 8.546E6 | 1 | 2390 | YS_1762.raw | 1 | PEAKS DB |
| LLLPGELA | 26.02 | 824.5007 | 8 | -0.7 | 825.5074 | 45.59 | 9.0747E4 | 1 | 21837 | YS_1762.raw | 1 | PEAKS DB |
| PGPMGPM | 30.34 | 685.2927 | 7 | -0.6 | 686.2996 | 14.6 | 1.1417E7 | 1 | 7090 | YS_1762.raw | 2 | PEAKS DB |
| AVFPSIV | 31.13 | 731.4218 | 7 | 0.6 | 732.4294 | 44.7 | 1.7249E5 | 1 | 21376 | YS_1762.raw | 1 | PEAKS DB |

**Table S1 continued.** Peptide information

| Peptide | -10lgP | Mass | Length | ppm | m/z | RT | Area 1 | Fraction | Scan | Source File | #Feature | Found By |
|---|---|---|---|---|---|---|---|---|---|---|---|---|
| YPPQTPL | 27.87 | 814.4225 | 7 | -0.4 | 815.4294 | 17.78 | 2.2569E7 | 1 | 8787 | YS_1762.raw | 1 | PEAKS DB |
| VGPVGPS | 33.11 | 611.3279 | 7 | -0.1 | 612.3351 | 7.72 | 2.5563E8 | 1 | 3732 | YS_1762.raw | 1 | PEAKS DB |
| NDPFIDL | 25.64 | 832.3967 | 7 | -0.6 | 833.4034 | 44.73 | 2.8175E4 | 1 | 21441 | YS_1762.raw | 1 | PEAKS DB |
| PGLPGMP | 30.88 | 667.3363 | 7 | -0.7 | 668.3431 | 38.54 | 2.8531E7 | 1 | 18520 | YS_1762.raw | 1 | PEAKS DB |
| IGFAGAP | 20.71 | 631.3329 | 7 | -5.5 | 316.672 | 41.93 | 3.2424E5 | 1 | 20333 | YS_1762.raw | 1 | PEAKS DB |
| GPAGPIG | 29.76 | 567.3016 | 7 | -0.8 | 568.3085 | 9.3 | 3.3773E7 | 1 | 4482 | YS_1762.raw | 2 | PEAKS DB |
| GPAGPLG | 29.76 | 567.3016 | 7 | -0.8 | 568.3085 | 9.3 | 3.3773E7 | 1 | 4482 | YS_1762.raw | 2 | PEAKS DB |
| PGIPGPM | 34.09 | 667.3363 | 7 | 0 | 668.3436 | 36.82 | 3.7589E7 | 1 | 17611 | YS_1762.raw | 2 | PEAKS DB |
| PGPSGPR | 28.97 | 666.3449 | 7 | -0.6 | 334.1795 | 5.33 | 5.1175E6 | 1 | 2635 | YS_1762.raw | 1 | PEAKS DB |
| GPVGPVG | 34.38 | 581.3173 | 7 | 1.6 | 582.3254 | 9.02 | 5.3054E7 | 1 | 4357 | YS_1762.raw | 1 | PEAKS DB |
| IPIDGDL | 23.49 | 741.3909 | 7 | -0.1 | 742.3981 | 28.24 | 8.269E5 | 1 | 13669 | YS_1762.raw | 1 | PEAKS DB |
| GLM(+15.99)GPAG | 24.42 | 617.2843 | 7 | -5.7 | 618.2881 | 8.61 | 9.0147E8 | 1 | 4465 | YS_1762.raw | 1 | PEAKS DB |
| GYPGPAG | 23.57 | 617.2809 | 7 | -0.1 | 618.2881 | 8.61 | 9.0147E8 | 1 | 4198 | YS_1762.raw | 1 | PEAKS DB |
| VGPGMP | 22.97 | 556.2679 | 6 | -0.5 | 557.2749 | 55.14 | 1.009E6 | 1 | 26072 | YS_1762.raw | 2 | PEAKS DB |
| GPGGGP | 20.18 | 440.2019 | 6 | 9.3 | 441.2133 | 1.64 | 1.0676E5 | 1 | 697 | YS_1762.raw | 1 | PEAKS DB |
| PGAGIP | 22.22 | 510.2802 | 6 | 0.5 | 511.2877 | 8.74 | 1.0682E9 | 1 | 4187 | YS_1762.raw | 1 | PEAKS DB |
| GPAGPI | 25.74 | 510.2802 | 6 | 0.5 | 511.2877 | 8.74 | 1.0698E9 | 1 | 4448 | YS_1762.raw | 3 | PEAKS DB |
| GPAGPL | 25.74 | 510.2802 | 6 | 0.5 | 511.2877 | 8.74 | 1.0698E9 | 1 | 4448 | YS_1762.raw | 3 | PEAKS DB |
| GPPGIA | 25.15 | 510.2802 | 6 | -0.3 | 511.2873 | 6.42 | 1.2822E8 | 1 | 3073 | YS_1762.raw | 1 | PEAKS DB |
| GPPGLA | 25.15 | 510.2802 | 6 | -0.3 | 511.2873 | 6.42 | 1.2822E8 | 1 | 3073 | YS_1762.raw | 1 | PEAKS DB |
| IGNERF | 20.68 | 734.3711 | 6 | 0.2 | 368.1929 | 3.35 | 1.3911E6 | 1 | 1554 | YS_1762.raw | 1 | PEAKS DB |
| IGM(+15.99)LGP | 20.15 | 602.3098 | 6 | -5.7 | 603.3136 | 18.69 | 1.487E8 | 1 | 8940 | YS_1762.raw | 1 | PEAKS DB |
| PGSPPP | 23.26 | 550.2751 | 6 | 0.4 | 551.2826 | 38.72 | 1.7579E5 | 1 | 18833 | YS_1762.raw | 1 | PEAKS DB |
| GPPGSP | 21.57 | 510.2438 | 6 | 1 | 511.2516 | 38.84 | 1.9293E6 | 1 | 18660 | YS_1762.raw | 2 | PEAKS DB |

**Table S1 continued.** Peptide information

| Peptide | -10lgP | Mass | Length | ppm | m/z | RT | Area 1 | Fraction | Scan | Source File | #Feature | Found By |
|---|---|---|---|---|---|---|---|---|---|---|---|---|
| GPTGEL | 21.46 | 572.2806 | 6 | -0.7 | 573.2875 | 1.87 | 1.9575E7 | 1 | 953 | YS_1762.raw | 1 | PEAKS DB |
| IGIGIS | 24.63 | 558.3377 | 6 | -1.1 | 559.3444 | 32.85 | 1.9743E8 | 1 | 16072 | YS_1762.raw | 1 | PEAKS DB |
| GLPGAP | 22.76 | 510.2802 | 6 | 2.2 | 511.2885 | 10.1 | 1.9907E7 | 1 | 4891 | YS_1762.raw | 1 | PEAKS DB |
| PGPMGL | 24.95 | 570.2836 | 6 | -0.1 | 571.2908 | 18.69 | 2.3095E6 | 1 | 9118 | YS_1762.raw | 1 | PEAKS DB |
| GFPGPR | 25.48 | 629.3285 | 6 | 0 | 315.6715 | 0.95 | 2.4416E6 | 1 | 513 | YS_1762.raw | 1 | PEAKS DB |
| GLPGTP | 21.51 | 540.2908 | 6 | -0.6 | 541.2977 | 9.75 | 2.6329E7 | 1 | 4717 | YS_1762.raw | 1 | PEAKS DB |
| GIPGTP | 21.51 | 540.2908 | 6 | -0.6 | 541.2977 | 9.75 | 2.6329E7 | 1 | 4717 | YS_1762.raw | 1 | PEAKS DB |
| GPPGIP | 25.72 | 536.2958 | 6 | 2.3 | 537.3043 | 11.56 | 2.6975E6 | 1 | 5650 | YS_1762.raw | 1 | PEAKS DB |
| GPPGLP | 25.72 | 536.2958 | 6 | 2.3 | 537.3043 | 11.56 | 2.6975E6 | 1 | 5650 | YS_1762.raw | 1 | PEAKS DB |
| QPAGPI | 22.89 | 581.3173 | 6 | 0.1 | 582.3246 | 10.97 | 2.7308E6 | 1 | 5370 | YS_1762.raw | 1 | PEAKS DB |
| GETGAP | 22.08 | 530.2336 | 6 | 1.7 | 531.2418 | 54.93 | 2.7481E6 | 1 | 25275 | YS_1762.raw | 1 | PEAKS DB |
| TGPLGI | 24.8 | 556.322 | 6 | 0.6 | 557.3297 | 19.77 | 2.7502E6 | 1 | 9596 | YS_1762.raw | 1 | PEAKS DB |
| PGPGMP | 28.37 | 554.2523 | 6 | 0.4 | 555.2598 | 28.74 | 2.7661E8 | 1 | 13926 | YS_1762.raw | 2 | PEAKS DB |
| GPVGPM | 24.74 | 556.2679 | 6 | 1.2 | 557.2758 | 10.62 | 2.9012E8 | 1 | 5136 | YS_1762.raw | 1 | PEAKS DB |
| GPMGPI | 26 | 570.2836 | 6 | 1 | 571.2914 | 17.72 | 3.2975E6 | 1 | 8610 | YS_1762.raw | 1 | PEAKS DB |
| TSPGPQ | 22.34 | 585.2758 | 6 | 3.1 | 586.2849 | 53.39 | 3.9427E4 | 1 | 25499 | YS_1762.raw | 2 | PEAKS DB |
| GPVGPV | 24.6 | 524.2958 | 6 | -1 | 525.3026 | 9.56 | 4.2233E7 | 1 | 4624 | YS_1762.raw | 1 | PEAKS DB |
| IDFPEF | 21.59 | 766.3537 | 6 | 1.3 | 767.362 | 48.79 | 4.4575E5 | 1 | 23295 | YS_1762.raw | 1 | PEAKS DB |
| GPAGPQ | 26.31 | 525.2547 | 6 | 0.2 | 526.2621 | 33.6 | 5.1652E6 | 1 | 16219 | YS_1762.raw | 2 | PEAKS DB |
| GPSGPQ | 25.04 | 541.2496 | 6 | -1.3 | 542.2562 | 38.3 | 5.2775E6 | 1 | 20060 | YS_1762.raw | 2 | PEAKS DB |
| GPVGPS | 22.66 | 512.2595 | 6 | -0.5 | 513.2665 | 42.17 | 5.4236E6 | 1 | 21553 | YS_1762.raw | 1 | PEAKS DB |
| TGTPGA | 20.2 | 502.2387 | 6 | -1.6 | 503.2452 | 52.39 | 5.7181E5 | 1 | 25714 | YS_1762.raw | 1 | PEAKS DB |
| GPAGAP | 27.46 | 468.2332 | 6 | 0.5 | 469.2407 | 31.08 | 7.1278E6 | 1 | 15028 | YS_1762.raw | 2 | PEAKS DB |
| FSGLDQ | 25.1 | 665.302 | 6 | -0.1 | 666.3092 | 16.08 | 7.1683E6 | 1 | 7827 | YS_1762.raw | 2 | PEAKS DB |

**Table S1 continued.** Peptide information

| Peptide | -10lgP | Mass | Length | ppm | m/z | RT | Area 1 | Fraction | Scan | Source File | #Feature | Found By |
|---|---|---|---|---|---|---|---|---|---|---|---|---|
| GFSGAP | 20.86 | 534.2438 | 6 | -0.1 | 535.251 | 50.55 | 7.6516E4 | 1 | 24362 | YS_1762.raw | 1 | PEAKS DB |
| GLMGIP | 20.02 | 586.3149 | 6 | 0.1 | 587.3222 | 33.67 | 8.7773E5 | 1 | 16158 | YS_1762.raw | 1 | PEAKS DB |
| PGSRGP | 20.18 | 569.2921 | 6 | 0.8 | 570.2999 | 54.18 | 9.1566E4 | 1 | 26045 | YS_1762.raw | 1 | PEAKS DB |
| GPIGP | 23.13 | 439.243 | 5 | 0.5 | 440.2505 | 1.55 | 1.1682E8 | 1 | 844 | YS_1762.raw | 1 | PEAKS DB |
| GPLGP | 23.13 | 439.243 | 5 | 0.5 | 440.2505 | 1.55 | 1.1682E8 | 1 | 844 | YS_1762.raw | 1 | PEAKS DB |
| GNSGP | 20.69 | 430.1812 | 5 | 0.3 | 431.1886 | 1.11 | 1.3001E4 | 1 | 523 | YS_1762.raw | 1 | PEAKS DB |
| TSAGP | 28.05 | 431.2016 | 5 | 0.7 | 432.2092 | 50.53 | 1.3645E6 | 1 | 24121 | YS_1762.raw | 2 | PEAKS DB |
| PGSPG | 21.36 | 413.191 | 5 | 1.7 | 414.199 | 8.7 | 1.4744E7 | 1 | 4055 | YS_1762.raw | 1 | PEAKS DB |
| GIAGL | 25.76 | 429.2587 | 5 | 0.7 | 430.2663 | 15.33 | 1.5998E6 | 1 | 7474 | YS_1762.raw | 1 | PEAKS DB |
| GLAGL | 25.76 | 429.2587 | 5 | 0.7 | 430.2663 | 15.33 | 1.5998E6 | 1 | 7474 | YS_1762.raw | 1 | PEAKS DB |
| QPGSP | 22.96 | 484.2281 | 5 | 1.4 | 485.2361 | 40.5 | 1.7318E6 | 1 | 19437 | YS_1762.raw | 1 | PEAKS DB |
| EGAKF | 21.07 | 550.2751 | 5 | 0.4 | 551.2826 | 38.72 | 1.7579E5 | 1 | 18559 | YS_1762.raw | 1 | PEAKS DB |
| LSGPG | 20.12 | 429.2223 | 5 | 0.4 | 215.6185 | 59.66 | 1.8371E5 | 1 | 28293 | YS_1762.raw | 1 | PEAKS DB |
| LGAGP | 22.97 | 413.2274 | 5 | 1.2 | 414.2352 | 37.72 | 1.8736E6 | 1 | 18121 | YS_1762.raw | 2 | PEAKS DB |
| GLPGM | 20.95 | 473.2308 | 5 | 0.7 | 474.2384 | 15.23 | 1.908E6 | 1 | 7456 | YS_1762.raw | 1 | PEAKS DB |
| PVGPM | 20.79 | 499.2465 | 5 | 1.6 | 500.2545 | 9.23 | 1.9699E7 | 1 | 4359 | YS_1762.raw | 1 | PEAKS DB |
| LPIID | 22.1 | 569.3424 | 5 | -0.1 | 570.3496 | 29 | 2.8153E6 | 1 | 13984 | YS_1762.raw | 2 | PEAKS DB |
| IPLLD | 22.1 | 569.3424 | 5 | -0.1 | 570.3496 | 29 | 2.8153E6 | 1 | 13984 | YS_1762.raw | 2 | PEAKS DB |
| GLPGE | 20.14 | 471.2329 | 5 | 1.1 | 472.2407 | 1.21 | 3.3376E7 | 1 | 584 | YS_1762.raw | 1 | PEAKS DB |
| GIPGE | 20.14 | 471.2329 | 5 | 1.1 | 472.2407 | 1.21 | 3.3376E7 | 1 | 584 | YS_1762.raw | 1 | PEAKS DB |
| PPGAP | 24.64 | 437.2274 | 5 | 1.4 | 438.2353 | 25.46 | 3.5849E7 | 1 | 12375 | YS_1762.raw | 1 | PEAKS DB |
| VLIPK | 20.2 | 568.3948 | 5 | -0.7 | 285.2045 | 7.59 | 4.3155E6 | 1 | 3690 | YS_1762.raw | 1 | PEAKS DB |
| VALGI | 27.03 | 471.3057 | 5 | 0.9 | 472.3134 | 40.3 | 4.5081E8 | 1 | 19343 | YS_1762.raw | 1 | PEAKS DB |
| IGIGI | 24.8 | 471.3057 | 5 | 0 | 472.3129 | 37.68 | 4.5081E8 | 1 | 18043 | YS_1762.raw | 1 | PEAKS DB |

**Table S1 continued.** Peptide information

| Peptide | -10lgP | Mass | Length | ppm | m/z | RT | Area 1 | Fraction | Scan | Source File | #Feature | Found By |
|---|---|---|---|---|---|---|---|---|---|---|---|---|
| GLIGL | 24.01 | 471.3057 | 5 | 0 | 472.3129 | 37.68 | 4.5081E8 | 1 | 18303 | YS_1762.raw | 1 | PEAKS DB |
| PGAPP | 24.27 | 437.2274 | 5 | 0.1 | 438.2347 | 36.75 | 4.7165E7 | 1 | 17545 | YS_1762.raw | 2 | PEAKS DB |
| VSGPQ | 21.95 | 486.2438 | 5 | 1.4 | 487.2518 | 40.3 | 4.7378E6 | 1 | 19346 | YS_1762.raw | 1 | PEAKS DB |
| GLIGP | 20.81 | 455.2744 | 5 | -0.2 | 456.2815 | 15.78 | 4.7787E6 | 1 | 7639 | YS_1762.raw | 1 | PEAKS DB |
| GDIGM | 24.11 | 491.205 | 5 | -0.8 | 492.2119 | 4.76 | 5.4316E7 | 1 | 2529 | YS_1762.raw | 1 | PEAKS DB |
| PILIP | 20.07 | 551.3683 | 5 | 0.3 | 552.3757 | 40.25 | 5.4657E4 | 1 | 19277 | YS_1762.raw | 1 | PEAKS DB |
| IPPAP | 23.09 | 493.29 | 5 | -3.7 | 494.2954 | 37.72 | 5.7774E5 | 1 | 18098 | YS_1762.raw | 1 | PEAKS DB |
| GSPGA | 22.3 | 387.1754 | 5 | 0.5 | 388.1829 | 45.33 | 6.2634E6 | 1 | 21696 | YS_1762.raw | 2 | PEAKS DB |
| APGSP | 20.07 | 427.2067 | 5 | -1.7 | 428.2132 | 50.9 | 6.6769E3 | 1 | 24308 | YS_1762.raw | 1 | PEAKS DB |
| VGSP | 23.51 | 358.1852 | 4 | 0.5 | 359.1927 | 51.78 | 1.0024E7 | 1 | 24707 | YS_1762.raw | 3 | PEAKS DB |
| TPYP | 28.07 | 476.2271 | 4 | 0.3 | 477.2345 | 0.23 | 1.034E7 | 1 | 172 | YS_1762.raw | 1 | PEAKS DB |
| GPSG | 22.49 | 316.1383 | 4 | 0.9 | 317.1458 | 53.16 | 1.0725E5 | 1 | 25291 | YS_1762.raw | 4 | PEAKS DB |
| IGM(+15.99)L | 21.55 | 448.2356 | 4 | -7.9 | 449.2393 | 16.61 | 1.0869E6 | 1 | 8110 | YS_1762.raw | 1 | PEAKS DB |
| LGM(+15.99)I | 21.55 | 448.2356 | 4 | -7.9 | 449.2393 | 16.61 | 1.0869E6 | 1 | 8110 | YS_1762.raw | 1 | PEAKS DB |
| AGPR | 21.85 | 399.223 | 4 | 0.1 | 200.6188 | 33.24 | 1.1026E7 | 1 | 16050 | YS_1762.raw | 5 | PEAKS DB |
| SDGL | 27.18 | 390.175 | 4 | 0.9 | 391.1827 | 58.24 | 1.1268E5 | 1 | 27809 | YS_1762.raw | 1 | PEAKS DB |
| VIIK | 23.46 | 471.342 | 4 | -0.5 | 236.6782 | 0.64 | 1.1728E7 | 1 | 169 | YS_1762.raw | 1 | PEAKS DB |
| HHGL | 23.35 | 462.2339 | 4 | 7.4 | 463.2446 | 25.62 | 1.2282E4 | 1 | 12470 | YS_1762.raw | 1 | PEAKS DB |
| FDIG | 25.75 | 450.2114 | 4 | 0.1 | 451.2188 | 13.97 | 1.2523E7 | 1 | 6784 | YS_1762.raw | 1 | PEAKS DB |
| VGPI | 26.88 | 384.2372 | 4 | 0.8 | 385.2448 | 7.76 | 1.2556E8 | 1 | 3752 | YS_1762.raw | 3 | PEAKS DB |
| VGPL | 26.88 | 384.2372 | 4 | 0.8 | 385.2448 | 7.76 | 1.2556E8 | 1 | 3752 | YS_1762.raw | 3 | PEAKS DB |
| TLAP | 20.37 | 400.2322 | 4 | 0.1 | 401.2395 | 1.23 | 1.2696E6 | 1 | 486 | YS_1762.raw | 1 | PEAKS DB |
| PGAP | 24.9 | 340.1747 | 4 | 0 | 341.1819 | 59.58 | 1.2932E5 | 1 | 28263 | YS_1762.raw | 2 | PEAKS DB |
| TGIP | 25.03 | 386.2165 | 4 | 1.1 | 387.2242 | 58.37 | 1.39E5 | 1 | 27708 | YS_1762.raw | 1 | PEAKS DB |

**Table S1 continued.** Peptide information

| Peptide | -10lgP | Mass | Length | ppm | m/z | RT | Area 1 | Fraction | Scan | Source File | #Feature | Found By |
|---|---|---|---|---|---|---|---|---|---|---|---|---|
| TGLP | 25.03 | 386.2165 | 4 | 1.1 | 387.2242 | 58.37 | 1.39E5 | 1 | 27708 | YS_1762.raw | 1 | PEAKS DB |
| LGYP | 25.79 | 448.2322 | 4 | -1.4 | 449.2388 | 7.97 | 1.4216E6 | 1 | 3789 | YS_1762.raw | 1 | PEAKS DB |
| LGPY | 24.49 | 448.2322 | 4 | -1.4 | 449.2388 | 7.97 | 1.4216E6 | 1 | 3831 | YS_1762.raw | 1 | PEAKS DB |
| KVII | 21.01 | 471.342 | 4 | 0.5 | 472.3495 | 0.69 | 1.4345E6 | 1 | 450 | YS_1762.raw | 1 | PEAKS DB |
| VLIP | 26.74 | 440.2999 | 4 | 0 | 441.3071 | 20.89 | 1.5492E6 | 1 | 10177 | YS_1762.raw | 1 | PEAKS DB |
| KDAP | 23.45 | 429.2223 | 4 | 0.6 | 430.2299 | 34.17 | 1.6307E6 | 1 | 16198 | YS_1762.raw | 1 | PEAKS DB |
| LAPT | 22.08 | 400.2322 | 4 | -0.3 | 401.2393 | 5.53 | 1.7067E7 | 1 | 2947 | YS_1762.raw | 1 | PEAKS DB |
| KMGT | 23.12 | 435.2151 | 4 | -4.4 | 436.2205 | 0.35 | 1.8051E5 | 1 | 124 | YS_1762.raw | 1 | PEAKS DB |
| PGSM | 23.41 | 390.1573 | 4 | -0.7 | 391.1643 | 9.3 | 1.8238E7 | 1 | 4505 | YS_1762.raw | 1 | PEAKS DB |
| APGA | 20.35 | 314.159 | 4 | 1.2 | 315.1667 | 58.98 | 2.0288E6 | 1 | 28524 | YS_1762.raw | 1 | PEAKS DB |
| GLAG | 29.1 | 316.1747 | 4 | 0.5 | 317.1821 | 47.75 | 2.1714E6 | 1 | 22827 | YS_1762.raw | 1 | PEAKS DB |
| GIAG | 29.1 | 316.1747 | 4 | 0.5 | 317.1821 | 47.75 | 2.1714E6 | 1 | 22827 | YS_1762.raw | 1 | PEAKS DB |
| MSPL | 25.84 | 446.2199 | 4 | 0.2 | 447.2273 | 11.47 | 2.2173E7 | 1 | 5549 | YS_1762.raw | 1 | PEAKS DB |
| AAGT | 25.55 | 318.1539 | 4 | 0 | 319.1612 | 58.3 | 2.2196E4 | 1 | 27733 | YS_1762.raw | 1 | PEAKS DB |
| VGGF | 28.02 | 378.1903 | 4 | 0 | 379.1976 | 2.72 | 2.2661E6 | 1 | 1316 | YS_1762.raw | 1 | PEAKS DB |
| RGPA | 23.11 | 399.223 | 4 | 0.6 | 400.2305 | 54.89 | 2.2979E6 | 1 | 27047 | YS_1762.raw | 1 | PEAKS DB |
| RPGA | 20.11 | 399.223 | 4 | 0.6 | 400.2305 | 54.89 | 2.2979E6 | 1 | 24802 | YS_1762.raw | 1 | PEAKS DB |
| GLGM | 24.29 | 376.178 | 4 | 0.6 | 377.1855 | 2.32 | 2.3175E6 | 1 | 1026 | YS_1762.raw | 1 | PEAKS DB |
| MGPM | 26.81 | 434.1658 | 4 | 1.2 | 435.1736 | 1.45 | 2.3413E6 | 1 | 578 | YS_1762.raw | 1 | PEAKS DB |
| DGAP | 20.12 | 358.1488 | 4 | 0.3 | 359.1562 | 54.74 | 2.3658E5 | 1 | 25756 | YS_1762.raw | 1 | PEAKS DB |
| PGIP | 23.05 | 382.2216 | 4 | -0.3 | 383.2288 | 0.01 | 2.3728E7 | 1 | 573 | YS_1762.raw | 2 | PEAKS DB |
| PGLP | 23.05 | 382.2216 | 4 | -0.3 | 383.2288 | 0.01 | 2.3728E7 | 1 | 573 | YS_1762.raw | 2 | PEAKS DB |
| RIMQ | 23.71 | 546.2948 | 4 | -5.5 | 547.2991 | 7.72 | 2.4251E6 | 1 | 3781 | YS_1762.raw | 1 | PEAKS DB |
| IGGT | 23.59 | 346.1852 | 4 | 0.6 | 347.1927 | 58.53 | 2.5717E5 | 1 | 28097 | YS_1762.raw | 1 | PEAKS DB |

**Table S1 continued.** Peptide information

| Peptide | -10lgP | Mass | Length | ppm | m/z | RT | Area 1 | Fraction | Scan | Source File | #Feature | Found By |
|---|---|---|---|---|---|---|---|---|---|---|---|---|
| DAGP | 23.1 | 358.1488 | 4 | 0.4 | 359.1562 | 48.29 | 2.5822E5 | 1 | 23038 | YS_1762.raw | 2 | PEAKS DB |
| VGPF | 29.07 | 418.2216 | 4 | -0.8 | 419.2285 | 15.26 | 2.6167E8 | 1 | 7602 | YS_1762.raw | 1 | PEAKS DB |
| AVAV | 25.7 | 358.2216 | 4 | 0.1 | 359.2289 | 0.83 | 2.6527E7 | 1 | 1017 | YS_1762.raw | 2 | PEAKS DB |
| MPGA | 23.29 | 374.1624 | 4 | -0.2 | 375.1696 | 46.05 | 2.6788E6 | 1 | 22010 | YS_1762.raw | 2 | PEAKS DB |
| ATPA | 20.79 | 358.1852 | 4 | -0.2 | 359.1924 | 58.95 | 2.9336E6 | 1 | 27830 | YS_1762.raw | 1 | PEAKS DB |
| KSIP | 21.46 | 443.2744 | 4 | 0.5 | 444.2819 | 1.15 | 3.0441E5 | 1 | 737 | YS_1762.raw | 1 | PEAKS DB |
| MAGL | 24.51 | 390.1937 | 4 | -7.9 | 391.1979 | 48.57 | 3.2441E4 | 1 | 23032 | YS_1762.raw | 1 | PEAKS DB |
| IVFD | 20.91 | 492.2584 | 4 | 1.5 | 493.2664 | 48.86 | 3.2597E4 | 1 | 23305 | YS_1762.raw | 1 | PEAKS DB |
| TAAT | 20.55 | 362.1801 | 4 | 1.9 | 363.1881 | 58.28 | 3.5165E4 | 1 | 27744 | YS_1762.raw | 1 | PEAKS DB |
| SVPP | 21 | 398.2165 | 4 | -0.1 | 399.2238 | 0.01 | 3.5219E7 | 1 | 281 | YS_1762.raw | 1 | PEAKS DB |
| GPGS | 22.54 | 316.1383 | 4 | 1.5 | 317.146 | 48.59 | 3.5524E6 | 1 | 23302 | YS_1762.raw | 7 | PEAKS DB |
| AGAP | 24.55 | 314.159 | 4 | 0.5 | 315.1664 | 42.42 | 3.7422E6 | 1 | 20333 | YS_1762.raw | 1 | PEAKS DB |
| PGPF | 25.84 | 416.2059 | 4 | -0.3 | 417.2131 | 10.65 | 3.7878E6 | 1 | 5183 | YS_1762.raw | 1 | PEAKS DB |
| RGAP | 23.63 | 399.223 | 4 | 0.6 | 400.2305 | 54.89 | 4.3481E6 | 1 | 24838 | YS_1762.raw | 2 | PEAKS DB |
| VPGV | 22.12 | 370.2216 | 4 | 0 | 371.2289 | 0.01 | 4.5301E6 | 1 | 60 | YS_1762.raw | 1 | PEAKS DB |
| GVGI | 27.41 | 344.206 | 4 | -0.1 | 345.2132 | 0.13 | 4.5321E6 | 1 | 788 | YS_1762.raw | 2 | PEAKS DB |
| TGPV | 28.43 | 372.2009 | 4 | -0.3 | 373.208 | 41.76 | 5.0926E6 | 1 | 19936 | YS_1762.raw | 1 | PEAKS DB |
| IVVA | 21.31 | 400.2686 | 4 | 1 | 401.2762 | 1.21 | 5.1181E5 | 1 | 486 | YS_1762.raw | 1 | PEAKS DB |
| GIGI | 28.85 | 358.2216 | 4 | 1 | 359.2292 | 13.49 | 5.2426E6 | 1 | 6589 | YS_1762.raw | 1 | PEAKS DB |
| GLAM | 22.39 | 390.1937 | 4 | -8.8 | 391.1975 | 1.16 | 5.4468E6 | 1 | 695 | YS_1762.raw | 1 | PEAKS DB |
| VMVP | 22.68 | 444.2406 | 4 | -0.5 | 445.2477 | 8.93 | 5.571E5 | 1 | 4365 | YS_1762.raw | 1 | PEAKS DB |
| AGPA | 25.86 | 314.159 | 4 | 0.5 | 315.1664 | 48.87 | 5.7711E6 | 1 | 23345 | YS_1762.raw | 2 | PEAKS DB |
| VSGP | 25.87 | 358.1852 | 4 | 0.6 | 359.1927 | 0.26 | 6.0435E6 | 1 | 374 | YS_1762.raw | 3 | PEAKS DB |
| TGAP | 25.56 | 344.1696 | 4 | -0.2 | 345.1768 | 47.23 | 6.7319E5 | 1 | 22310 | YS_1762.raw | 1 | PEAKS DB |

**Table S1 continued.** Peptide information

| Peptide | -10lgP | Mass | Length | ppm | m/z | RT | Area 1 | Fraction | Scan | Source File | #Feature | Found By |
|---|---|---|---|---|---|---|---|---|---|---|---|---|
| TGPA | 24.11 | 344.1696 | 4 | -0.2 | 345.1768 | 47.23 | 6.7319E5 | 1 | 22003 | YS_1762.raw | 1 | PEAKS DB |
| AGLE | 20.06 | 388.1958 | 4 | 1.1 | 389.2035 | 53.32 | 6.8878E4 | 1 | 25532 | YS_1762.raw | 1 | PEAKS DB |
| FGPA | 24.9 | 390.1903 | 4 | -1.8 | 391.1969 | 51.33 | 7.1095E4 | 1 | 24449 | YS_1762.raw | 3 | PEAKS DB |
| PGSP | 22.85 | 356.1696 | 4 | -0.7 | 357.1766 | 23.6 | 7.1267E7 | 1 | 11427 | YS_1762.raw | 4 | PEAKS DB |
| IGPM | 26.86 | 416.2094 | 4 | 0.1 | 417.2167 | 9.62 | 7.3071E6 | 1 | 4701 | YS_1762.raw | 1 | PEAKS DB |
| LGPM | 26.86 | 416.2094 | 4 | 0.1 | 417.2167 | 9.62 | 7.3071E6 | 1 | 4701 | YS_1762.raw | 1 | PEAKS DB |
| VGAP | 32 | 342.1903 | 4 | 0.4 | 343.1977 | 25.73 | 7.5806E6 | 1 | 12503 | YS_1762.raw | 1 | PEAKS DB |
| TAGP | 27.36 | 344.1696 | 4 | -0.2 | 345.1768 | 47.23 | 7.9372E5 | 1 | 21316 | YS_1762.raw | 2 | PEAKS DB |
| ILGL | 30.12 | 414.2842 | 4 | 0.3 | 415.2916 | 30.58 | 8.1801E5 | 1 | 14782 | YS_1762.raw | 1 | PEAKS DB |
| AVAL | 24.26 | 372.2372 | 4 | 0.9 | 373.2448 | 8.23 | 8.35E6 | 1 | 4056 | YS_1762.raw | 1 | PEAKS DB |
| IGPF | 28.18 | 432.2372 | 4 | 0.1 | 433.2446 | 21.6 | 8.407E6 | 1 | 10447 | YS_1762.raw | 1 | PEAKS DB |
| LGPF | 28.18 | 432.2372 | 4 | 0.1 | 433.2446 | 21.6 | 8.407E6 | 1 | 10447 | YS_1762.raw | 1 | PEAKS DB |
| GAGR | 25.81 | 359.1917 | 4 | -8.1 | 360.1961 | 58.31 | 8.7913E4 | 1 | 27675 | YS_1762.raw | 1 | PEAKS DB |
| VYH | 22.28 | 417.2012 | 3 | 7.8 | 418.2117 | 50.12 | 1.0155E6 | 1 | 24815 | YS_1762.raw | 1 | PEAKS DB |
| GFL | 25.71 | 335.1845 | 3 | -1 | 336.1914 | 14.53 | 1.2579E7 | 1 | 6943 | YS_1762.raw | 1 | PEAKS DB |
| AGL | 31.33 | 259.1532 | 3 | 0.3 | 260.1606 | 43.5 | 1.6178E6 | 1 | 20839 | YS_1762.raw | 1 | PEAKS DB |
| MGL | 25.58 | 319.1566 | 3 | 0.8 | 320.1641 | 2.13 | 2.3248E6 | 1 | 976 | YS_1762.raw | 1 | PEAKS DB |
| LEF | 26.24 | 407.2056 | 3 | -0.9 | 408.2126 | 2.32 | 2.422E4 | 1 | 1106 | YS_1762.raw | 1 | PEAKS DB |
| GPF | 24.99 | 319.1532 | 3 | -0.4 | 320.1603 | 15.33 | 3.9464E6 | 1 | 7444 | YS_1762.raw | 1 | PEAKS DB |
| GFM | 23.14 | 353.1409 | 3 | 0.5 | 354.1484 | 4.58 | 3.9886E7 | 1 | 2045 | YS_1762.raw | 1 | PEAKS DB |
| TAP | 23.76 | 287.1481 | 3 | 1.7 | 288.1559 | 46.39 | 4.1026E4 | 1 | 22259 | YS_1762.raw | 1 | PEAKS DB |
| GPA | 28.67 | 243.1219 | 3 | 0.6 | 244.1293 | 39.33 | 4.2902E6 | 1 | 18889 | YS_1762.raw | 2 | PEAKS DB |
| MPI | 24.26 | 359.1879 | 3 | -1 | 360.1948 | 7.72 | 5.1251E7 | 1 | 3959 | YS_1762.raw | 2 | PEAKS DB |
| TGV | 28.09 | 275.1481 | 3 | -0.2 | 276.1554 | 59.46 | 5.2482E5 | 1 | 28167 | YS_1762.raw | 2 | PEAKS DB |

**Table S1 continued.** Peptide information

| Peptide | -10lgP | Mass | Length | ppm | m/z | RT | Area 1 | Fraction | Scan | Source File | #Feature | Found By |
|---|---|---|---|---|---|---|---|---|---|---|---|---|
| VGF | 27.05 | 321.1688 | 3 | -0.6 | 322.1759 | 2.98 | 5.2995E6 | 1 | 1379 | YS_1762.raw | 2 | PEAKS DB |
| KVP | 21.68 | 342.2267 | 3 | 1.3 | 343.2344 | 0.07 | 5.6444E5 | 1 | 159 | YS_1762.raw | 1 | PEAKS DB |
| DIF | 29.53 | 393.19 | 3 | 0.1 | 394.1973 | 21.48 | 6.4145E6 | 1 | 10399 | YS_1762.raw | 2 | PEAKS DB |
| GPR | 26.03 | 328.1859 | 3 | 0.3 | 329.1933 | 0.49 | 6.8983E6 | 1 | 231 | YS_1762.raw | 2 | PEAKS DB |
| EVG | 25.53 | 303.143 | 3 | 1.2 | 304.1506 | 58.77 | 6.9261E5 | 1 | 28275 | YS_1762.raw | 2 | PEAKS DB |
| GPK | 20.54 | 300.1797 | 3 | 0.4 | 301.1871 | 44.49 | 7.0543E4 | 1 | 21335 | YS_1762.raw | 1 | PEAKS DB |
| IPF | 28.73 | 375.2158 | 3 | -0.5 | 376.2229 | 21.09 | 9.2057E6 | 1 | 10453 | YS_1762.raw | 1 | PEAKS DB |
| GPS | 21.51 | 259.1168 | 3 | 0 | 260.1241 | 59 | 9.3007E5 | 1 | 28169 | YS_1762.raw | 1 | PEAKS DB |
| PGS | 21.42 | 259.1168 | 3 | 0 | 260.1241 | 59 | 9.3007E5 | 1 | 28270 | YS_1762.raw | 1 | PEAKS DB |
| GDDWDYIPLPRRH | 34.2 | 1638.7903 | 13 | 1.2 | 547.2714 | 29.28 | 0 | 1 | 14180 | YS_1762.raw | 0 | PEAKS DB |
| VGPA | 29.54 | 342.1903 | 4 | 0.5 | 343.1978 | 56.05 | 0 | 1 | 26698 | YS_1762.raw | 0 | PEAKS DB |
